# A Machine Learning Approach for Investigating Variable Importance in Relationship and Sexual Satisfaction: The Role of Interpersonal Mindfulness and Psychological Safety

**DOI:** 10.1111/jmft.70026

**Published:** 2025-04-24

**Authors:** Claudia Dias Martins, Rodrigo C. Vergara, Bassam Khoury

**Affiliations:** ^1^ Department of Educational and Counselling Psychology McGill University Montréal Québec Canada; ^2^ Departamento de Kinesiología Facultad de Artes y Educación Física, Universidad Metropolitana de Ciencias de la Educación Ñuñoa Chile; ^3^ Centro Nacional de Inteligencia Artificial (CENIA) Macul Chile

**Keywords:** interpersonal mindfulness, machine learning, psychological safety, relationship satisfaction, sexual satisfaction

## Abstract

Numerous studies have shown that mindfulness is positively associated with relationship and sexual satisfaction. However, most have examined the benefits of intrapersonal or trait mindfulness, rather than directly investigating interpersonal mindfulness or considering polyvagal theory. Our main objective was to determine the variable importance of interpersonal mindfulness and psychological safety for relationship and sexual satisfaction using random forests and regression trees and to explore the importance of demographics, social and couple‐related factors, and emotional wellbeing in this analysis. 356 adults in committed romantic relationships were recruited for a self‐report survey. Results suggested that mindfulness in couple relationships, psychological safety, conflict strategies, and depression symptoms were of top importance for relationship and sexual satisfaction. Limitations and future directions involving dyadic data and physiological measures were discussed. The findings will inform the development of interpersonal mindfulness‐ and polyvagal‐based interventions aimed at promoting safety and stability in relationships while enhancing personal wellbeing.

## 1 Introduction

In the field of couples research, relationship and sexual satisfaction appear to be consistent predictors of personal wellbeing, physical and mental health, mortality, job performance, and children's wellbeing (Carr and Springer 2010; Davison et al. 2009; Sánchez‐Fuentes et al. 2014; Dush and Amato 2005; Joel et al. 2020; Proulx et al. 2007). Given the numerous well‐documented benefits of enhanced relationship and sexual satisfaction, extensive research has been devoted to studying their predictors (for reviews, see Sánchez‐Fuentes et al. 2014; Karney and Bradbury 1995; Le et al. 2010). Building on this foundation, researchers are currently employing innovative techniques to consolidate findings and gain deeper insights into the most important predictors. For instance, Joel et al. (2020) applied a machine‐learning technique known as random forests to data from 43 longitudinal couples studies. Using this approach, the researchers identified the most reliable predictors of relationship satisfaction, categorizing them into relationship variables (i.e., perceived partner commitment, appreciation, sexual satisfaction, perceived partner satisfaction, and conflict), objective relationship variables (i.e., relationship length), and individual difference variables (i.e., life satisfaction, negative affect, depression, attachment anxiety, and avoidance).

Using a similar machine‐learning technique, Vowels et al. (2022) found that the most important predictors of sexual satisfaction included relationship variables (i.e., relationship satisfaction, dyadic desire, romantic love, sexual communication, perception of love, and desire), whereas the least important predictors included individual difference variables (i.e., gender, sexual orientation, children, religiosity, attitudes toward sexuality, and mental health). Interestingly, both studies found that demographic variables had relatively little impact on different types of satisfaction compared to relationship‐specific variables. Taken together, the studies highlight that while objective relationship variables and demographics may play a role, relationship‐specific variables and individual differences in wellbeing appear to be the strongest predictors of satisfaction (Joel et al. 2020; Vowels et al. 2022).

While previous research has established a strong link between relationship and sexual satisfaction, it is important to note that studies exploring the direction of this relationship have yielded mixed results. Some research suggests unidirectional effects, with relationship satisfaction sometimes predicting sexual satisfaction and, at other times, sexual satisfaction predicting relationship satisfaction (Vowels and Mark 2020; Yeh et al. 2006). However, there is also theoretical (Byers 2005) and empirical (McNulty et al. 2016; Quinn‐Nilas 2020a) support for bidirectional effects. Consequently, it is of interest to consider relationship satisfaction and sexual satisfaction as interconnected yet distinct constructs.

Despite limitations surrounding self‐report measures, the constructs of relationship satisfaction and sexual satisfaction are both commonly used as outcome measures in the evaluation of clinical intervention effectiveness (McClelland 2011; Wood et al. 2005). By continuing to investigate the predictors of each type of satisfaction, researchers will be able to identify treatment targets and fine‐tune the development of clinical interventions aimed at improving relational wellbeing. Given the growing interest in mindfulness interventions and insights from polyvagal theory which could benefit couples (Atkinson 2013; Bradford and Johnson 2023; Fox 2024; Porges 2017; Ryland et al. 2022; Siegel 2014; Winter et al. 2021), this paper aims to explore the relative importance of variables contributing to both relationship and sexual satisfaction with a particular focus on variables associated with mindfulness and psychological safety.

### 1.1 The Role of Mindfulness

According to Theravada Buddhism, mindfulness derives from the ancient Pāli word “sati.” Originally defined as memory, it evolved to encapsulate awareness, attention, and recollection of physical, sensory, and psychological experiences occurring in the present (Bodhi 2011; Olendzki 2014). In Western medicine and psychological science, research on mindfulness and its contemporary conceptualizations has grown exponentially over the last four decades (Khoury et al. 2017). Mindfulness is viewed as a form of directed, intentional and nonjudgmental attention to the present, cultivated through consistent personal practice, psychotherapeutic interventions incorporating Buddhist practices (e.g., breathing, mindful eating and walking, body scanning, yoga, and meditation) and psychoeducation in groups/individually (Khoury et al. 2017). Modern conceptualizations distinguish mindfulness as both a *state* induced during mindfulness practices (Lau et al. 2006) and a *trait* or enduring disposition to be mindful in everyday life which can be strengthened through intensive training (Baer et al. 2006; Brown and Ryan 2003; Kiken et al. 2015). While it is commonly studied as an intrapersonal skill (mindfulness with self), theoretical and empirical evidence suggest it also functions as an interpersonal skill (mindfulness with others, Khoury 2018) applicable within romantic relationships (mindfulness in couple relationships, McGill et al. 2022).

Emerging definitions and psychometric measures of interpersonal mindfulness expand beyond intrapersonal, or trait, mindfulness by integrating elements of awareness relevant to interpersonal interactions (Khoury 2018; Khoury et al. 2022; Pratscher et al. 2019) and couple relationships (Daks et al. 2021; Kimmes et al. 2018; McGill et al. 2022). Interpersonal mindfulness involves a combination of: (1) Listening attentively to others; (2) present‐centered awareness of emotions experienced by self and others during interactions; (3) openness, acceptance, and receptivity to others’ thoughts and feelings; (4) self‐regulation, marked by low emotional and behavioral reactivity and low automaticity in responses to the everyday behavior of others; and (5) compassion for self and others (Duncan et al. 2009). Although initially conceptualized in the context of parent–child relationships, this framework is applicable to broader relational contexts. Given that interpersonal relationships substantially influence physical and mental wellbeing and buffer against stressors (Agnew and South 2014; Henry et al. 2015; Holt‐Lunstad et al. 2010; Newman and Roberts 2013), it is important to explore the unique benefits of interpersonal mindfulness in couples.

Nevertheless, reviews of past research highlight the predominant focus on the positive association between facets of intrapersonal, or trait, mindfulness, and relationship and sexual satisfaction (Atkinson 2013; Kozlowski 2013; McGill et al. 2016; Quinn‐Nilas 2020b). Trait mindfulness and meditation experience appear to enhance relational outcomes by improving emotion regulation, empathy, compassion, communication, stress management, sexual wellbeing, and fostering secure attachment (Atkinson 2013). Furthermore, researchers have uncovered specific mechanisms through which mindfulness influences relationship satisfaction. These include facilitating intimacy development and perceived responsiveness during vulnerable discussions between partners (Adair et al. 2018; Khalifian and Barry 2021), promoting acceptance of partner imperfections (Kappen et al. 2018), encouraging positive conflict resolution styles and closeness (Gesell et al. 2020), and mitigating negative affect and the consequences of daily dips in satisfaction (Don and Algoe 2020; May et al. 2020). Prior research has also identified mindfulness as a moderator between conflict resolution and sexual satisfaction and as predictor of sexual satisfaction mediated by emotion regulation and cognitive distraction (Newcombe and Weaver 2016; Pepping et al. 2018; Smedley et al. 2021).

While research on the advantages of intrapersonal mindfulness for romantic relationships is growing, there remains a shortage of studies directly exploring the significance of interpersonal mindfulness in this context (exceptions include Khorasani et al. 2021; Kimmes et al. 2020). To address shortcomings in the conceptualization of interpersonal mindfulness, particularly within couple relationships, McGill et al. (2022) developed a multi‐dimensional and context‐specific measure including eight facets–nonjudging, patience, beginner's mind, trust of self, nonstriving, acceptance, letting go, and noticing (self and others). Compared to alternatives such as the Relationship Mindfulness Measure and the Attentive Awareness in Relationships Scale (Daks et al. 2021; Kimmes et al. 2018), McGill et al. (2022) position the 31‐item Mindfulness in Couple Relationships Scale (MCRS) as comprehensive and theory‐informed. It is rooted in Kabat‐Zinn's. (2009) definition of core mindfulness attitudes as well as Karremans et al.'s (2017) conceptualization of mindfulness in couples as an intentional and conscious effort to be present and accepting with one's own feelings and emotions and to recognize how these moment‐to‐moment experiences may impact one's partner and the relationship between them.

As a novel measure, the MCRS (McGill et al. 2022) offers researchers the opportunity to study mindfulness in a context where closeness and intimacy are experienced differently than other interpersonal relationships, while also exploring how specific subscales contribute to various outcomes. Since romantic relationships inherently depend on interpersonal connection, understanding the interpersonal aspects of mindfulness that contribute to positive relationships is essential for individuals and couples seeking to enhance relational wellbeing and, by extension, personal wellbeing. Having administered measures of both intrapersonal and interpersonal mindfulness in the current study, it is possible to make comparisons regarding their relative importance for adults in romantic relationships. As researchers continue to investigate whether manualized mindfulness and compassion training is as effective as stand‐alone practices in couples therapy, examining the multiple dimensions of interpersonal mindfulness will provide valuable insights. This, in turn, will help tailor mindfulness and compassion exercises (e.g., breathing, loving kindness meditation, mindful communication) to better support de‐escalation, mutual acceptance, and thoughtful discussions among partners (Lebow and Snyder 2022; Siegel 2014; Winter et al. 2021).

### 1.2 The Role of Psychological Safety

Grounded in neurophysiology, psychology, and evolutionary theory, Porges' Polyvagal Theory (PVT; 1995, 2011) provides an avenue for understanding how mindfulness in relationships can foster coregulation and attunement, cultivating a shared sense of calm, safety, and enduring pair‐bonds. PVT suggests that psychological safety is experienced on a subconscious level through a process of ‘neuroception’ in which individuals’ autonomic nervous systems (ANS) evaluate a situation for safety or threat (Porges 2004). With a focus on the tenth cranial nerve (i.e., vagus), the theory posits that each of its two branches serve a different function (Porges 1995, 1998). When the ANS detects safety in the environment, the ventral vagal pathway is activated—inhibiting sympathetic excitation and promoting calm and prosocial behaviors such as social engagement and compassion (Kolacz et al. 2019; Porges 1998). From this state of physiological and psychological calm, cues of safety can be communicated through active listening, rich vocal prosody, open body posture, gentle gestures, mutual eye contact, and warm facial expressions (Porges 1998, 2011, 2017). Whereas when the ANS detects a threat, the dorsal vagal pathway is activated—inhibiting social engagement and mobilizing the sympathetic nervous system for defensive fight or flight behaviors (Kolacz et al. 2019). While this evolutionary mechanism may be adaptive in response to major threats, an otherwise dysregulated state can get in the way of constructive conflict resolution and reparation in relationships.

To better understand psychological safety, a recently developed self‐report measure known as the Neuroception of Psychological Safety Scale (NPSS; Morton et al. 2024) aims to capture these core aspects of social engagement, compassion, and bodily sensations. Within this framework, social engagement is the ability to feel accepted, understood, cared for, and open to self‐expression without fear of judgment (Morton et al. 2024). Compassion—the ability to feel empathetic, caring, and motivated to help—can be cultivated through meditation practices (e.g., loving kindness and compassion meditation) and is increasingly linked with self‐soothing abilities and the communication of safety in interpersonal contexts (Gilbert 2017; Gilbert 2020; Luberto et al. 2018). Finally, bodily sensations captures an internal state of calm (e.g., regulation of heart rate and breath, relaxation of face, body and stomach), which can also be linked with physiological processes of relaxation accessed through mindfulness, meditation, and regulated breathing practices (Bradford and Johnson 2023; Dana 2021; Gilbert 2020; Luberto et al. 2018; Porges and Carter 2017). Importantly, mindfulness skills are highly relevant to these core aspects of safety and have been proposed as a means of regulating the nervous system from within while recalibrating perceptions of safety versus threat in the environment (Porges and Carter 2017).

Given the depth of connection and vulnerability shared between romantic partners, Porges (2017) theorizes that feelings of safety serve as a key mechanism for bonding, even before physical contact and intimacy. Building on this, mindfulness interventions are suggested to create a foundation for psychological safety in couples therapy, facilitating the optimal exploration of relationship stressors from an emotionally regulated state (Siegel 2014). It is believed that with the support of a present therapist, and through mindfulness and somatic practices, couples can learn to down‐regulate and communicate cues of safety in a process of coregulation (Fox 2024; Ryland et al. 2022). While further research is needed, it appears that strategies enhancing interpersonal mindfulness and psychological safety may, in turn, help reduce destructive conflict strategies and foster the love and trust essential for closeness (Gesell et al. 2020; Porges 1998, 2011, 2017; Siegel 2014; Smedley et al. 2021).

### 1.3 Bridging Interpersonal Mindfulness and Psychological Safety Through Polyvagal Theory

Alongside the interpersonal mindfulness framework, PVT offers a novel approach to understanding the role of mindfulness in romantic relationships. There is an established link between interpersonal mindfulness and compassion (e.g., Duncan et al. 2009; Khoury et al. 2022) and between psychological safety and compassion (e.g., Gilbert 2017, 2020; Morton et al. 2024). This intersection of compassion presents a compelling case for an integrated approach. As discussed above, the use of mindfulness and compassion practices (e.g., breathing and loving kindness meditation) are suggested to not only help romantic partners become more interpersonally mindful and coregulated but also to support them in cultivating and communicating safety both verbally and non‐verbally (e.g., Gilbert 2020; Siegel 2014; Winter et al. 2021). While PVT has gained recognition as a protective tool in interpersonal relationship building and communication in organizational contexts and parent‐child or therapist‐client relationships (Cozolino 2006; Geller and Porges 2014; Morton et al. 2024; Porges and Dana 2018), its connections to mindfulness remain largely theoretical, and its application to romantic relationships requires further empirical investigation.

### 1.4 Research Question

The existing literature indicates positive associations between trait mindfulness and relational outcomes, yet the role of interpersonal mindfulness and psychological safety in romantic relationships remains inadequately explored. The current study evaluated the interplay between mindfulness, demographics, social and couple‐related factors, and emotional wellbeing among adults in romantic relationships. Our main objective was to assess the variable importance of interpersonal mindfulness and psychological safety for relationship and sexual satisfaction, as well as explore the relative importance of previously associated variables, namely, demographics, trait mindfulness, empathy, compassion, conflict strategies, negative emotional states, and difficulties in emotion regulation, within this analytical framework.

Based on previous literature, we hypothesized that these variables would all have some contribution to relationship and sexual satisfaction. However, we chose to assess the level of importance of each variable as an exploratory analysis. Building upon the interpersonal mindfulness framework and PVT, we further hypothesized that measures of interpersonal mindfulness and psychological safety would be of greater importance to satisfaction, especially in comparison to intrapersonal mindfulness. Considering the multitude of variables that appear to be implicated in the relationship between mindfulness and satisfaction, and the diverse mechanisms through which mindfulness influences relational outcomes, employing machine‐ learning techniques in the current study offered a systematic approach to identifying the relative importance of all variables of interest. The novel analytic approach was expected to deepen our understanding of the predictors of satisfaction (see Analysis Strategy for more details), with findings that could inform future research and contribute to the development and evaluation of interpersonal mindfulness‐ and polyvagal‐based interventions for diverse individuals and couples in romantic relationships.

## 2 Method

### 2.1 Participants

Participants (*N* = 356) were adults living across Canada, who have been in a romantic relationship with the same partner for at least 1 year and have been sexually active in this relationship. Those who did not complete the survey were excluded from the analysis, reducing the sample from 542 eligible participants to 357 (65.87%). Additionally, one participant who reached the end of the survey but had 31 missing values in the variables of interest was removed (refer to Supporting Information for descriptive statistics and frequency of missing values among variables of interest). 246 of the 356 participants were without any missing values in the measures of interest.

The retained sample was composed of 356 individuals between the ages of 18–85 (*M*_Age_ = 36.09; SD_Age_ = 13.17). At the time of data collection, 283 (79.49%) participants identified as women, 58 (16.29%) identified as men, 7 (1.97%) identified as gender variant/non‐conforming, 6 (1.69%) identified as trans, and 2 (0.56%) identified as other. Most individuals were either in a relationship—living apart (31.46%) or married (31.18%), followed by those in a relationship—living together (17.13%), long‐term cohabitation or common law (15.73%), or engaged (3.93%). Two participants (0.56%) selected “Prefer Not to Answer” for their relationship status. Relationship length varied from 12 to 900 months (*M* = 110, median = 58.5, SD = 127.93). Detailed demographics, including diversity in ethnicity, relationship type, and sexual orientation, are available in Table 1.

**Table 1:** Demographic results.

	Mean ± SD	Min	1st Qu.	Median	3rd Qu.	max
Age	36.09 ± 13.17	18	26	32	46	85
Years of education	18.39 ± 3.28	4	16	18	20	29
Relationship length (months)	110 ± 127.93	12	24	58.5	144	900
Total meditation practice hours	289.5 ± 962.34	0	0	8.68	112.50	9385.80
Ethnicity	**White**	**Black**	**Indigenous**	**Chinese**	**Asian (other than Chinese)**	**Mixed**
	74.16%	2.25%	1.12%	4.78%	8.15%	8.43%
Gender identity	**Woman**	**Man**	**Gender variant/nonconforming**	**Trans**	**Other**	
	79.49%	16.29%	1.97%	1.69%	0.56%	
Sexual orientation	**Heterosexual**	**Bisexual**	**Gay**	**Lesbian**	**Questioning**	**Other**
	65.73%	22.19%	0.84%	3.37%	2.25%	5.06%
Relationship status	**Living together**	**Living apart**	**Long‐term cohabitation or common law**	**Engaged**	**Married**	**Did not answer**
	17.13%	31.46%	15.73%	3.93%	31.18%	0.56%
Relationship type	**Fully monogamous**	**Consensually nonmonogamous**	**Other**	**Did not answer**		
	92.13%	6.18%	1.12%	0.56%		
Employment situation	**Full‐time employment**	**Part‐time employment (≤ 30 h per week)**	**Seasonal/tem‐porary employment**	**Other**	**Did not answer**	
	51.40%	20.51%	5.06%	10.39%	12.64%	

Abbreviations: Qu., quartile; SD, standard deviation.

### 2.2 Procedures

Ethics approval for the current study was obtained from the McGill University Research Ethics Board in October 2023. Participants were recruited via social media ads and posters on campus. Data collection took place between January 2024 and April 2024, and participants were offered $50 prizes based on a draw with a 1/10 winning rate. Participants provided informed consent before completing a battery of self‐report questionnaires on *LimeSurvey*. The questionnaires included sociodemographic data with detailed information regarding their social identities, objective elements of their romantic relationship, and meditation or mindfulness experience, as well as a host of measures detailed below. Participants were able to complete the study on any desktop computer, laptop, tablet, or mobile device with internet access.

### 2.3 Measures

Based on associations between variables noted in previous literature, three groups of measures were used for analyses in addition to the sociodemographic questionnaire. The first group included four measures of intrapersonal and interpersonal mindfulness, namely, the 15‐item Five‐Facet Mindfulness Questionnaire (FFMQ; Baer et al. 2012), Interpersonal Mindfulness Scale (IMS; Pratscher et al. 2019), Interpersonal Mindfulness Questionnaire (IMQ; Khoury et al. 2022), and Mindfulness in Couple Relationships Scale (MCRS; McGill et al. 2022). The second group included six social and couple‐related measures, namely, the Couples Satisfaction Index (CSI; Funk and Rogge 2007), New Sexual Satisfaction Scale ‐ Short (NSSS; Brouillard et al. 2020), Neuroception of Psychological Safety Scale (NPSS; Morton et al. 2024), Romantic Partner Conflict Scale (RPCS; Zacchilli et al. 2009), Compassion Scale (CS; Pommier et al. 2019), and Toronto Empathy Questionnaire (TEQ; Spreng* et al. 2009). As suggested by the developers of the NPSS (Morton et al. 2021), the original instructions of this measure were edited to specify the context (i.e., please rate how well the following statements describe your feelings during your experiences with your romantic partner over the past week). Finally, the third group included two measures (for a total of 12) regarding emotional wellbeing, namely, the Depression Anxiety Stress Scales (DASS; Henry and Crawford 2005) and Difficulties in Emotion Regulation Scale—Short Form (DERS; Kaufman et al. 2016). Internal consistencies were calculated using Cronbach's alpha and ranged from acceptable to outstanding overall (Meyers et al. 2017; see Table 2).

**Table 2:** Internal consistencies of instruments administered.

Instrument	Total scale reliability	Subscale reliability				
Mindfulness						
FFMQ	0.84	Observe	Describe	Acting with awareness	Nonjudging	Nonreactivity
		0.67	0.82	0.83	0.87	0.79
IMS	0.93	Presence	Awareness of self and others	Nonjudgmental acceptance	Nonreactivity	
		0.88	0.89	0.76	0.78	
IMQ		Being caught in the mind	Attention to and awareness of others	Body‐anchored presence	Mindful responding	
		0.89	0.94	0.80	0.69	
MCRS	0.93	Nonjudging	Patience	Beginner's mind	Trust of self	Nonstriving
		0.81	0.76	0.82	0.80	0.70
		Acceptance	Letting go	Noticing		
		0.77	0.79	0.73		
Social and couple‐related						
CSI	0.96					
NSSS	0.93	Ego‐centered	Partner/activity‐centered			
		0.89	0.88			
NPSS	0.96	Social engagement	Compassion	Bodily sensations		
		0.95	0.92	0.93		
RPCS		Compromise	Avoidance	Interactional reactivity	Separation	Domination
		0.95	0.90	0.85	0.89	0.91
		Submission				
		0.93				
CS	0.86	Kindness	Common humanity	Mindfulness	Indifference	
		0.82	0.69	0.74	0.75	
TEQ	0.88					
Emotional wellbeing						
DASS		Depression	Anxiety	Stress		
		0.91	0.87	0.88		
DERS	0.90	Strategies	Nonacceptance	Impulse	Goals	Awareness
		0.83	0.87	0.89	0.92	0.74
		Clarity				
		0.85				

*Note:* Internal consistencies, calculated via Cronbach's Alpha, of total and subscale scores of instruments used for data analyses.

Abbreviations: CS Compassion Scale; CSI Couples Satisfaction Index; DASS Depression Anxiety Stress Scales; DERS Difficulties in Emotion Regulation Scale (18‐item version); FFMQ, Five‐Facet Mindfulness Questionnaire (15‐item version); IMQ Interpersonal Mindfulness Questionnaire; IMS Interpersonal Mindfulness Scale; MCRS Mindfulness in Couple Relationships Scale; NSSS New Sexual Satisfaction Scale (12‐item version); NPSS Neuroception of Psychological Safety Scale; RPCS Romantic Partner Conflict Scale; TEQ Toronto Empathy Questionnaire.

Additionally, we measured participants’ meditation experience based on an aggregated amount of meditation practice hours. This amount was calculated using participants’ responses regarding (1) average number of minutes practiced per week, (2) number of months since beginning to practice, wherein responses between 0 and 0.99 specified either no practice or less than 1 month based on the percentage of days they practiced during the month, (3) number of meditation retreats attended, and (4) number of hours practiced during retreats attended.

### 2.4 Analysis Strategy

The analysis consisted of three steps: (1) variable screening, (2) confirmation of predictor significance, and (3) assessment of masking effects. Step 1 involved narrowing the number of potentially relevant predictors based on their variable importance (see below for more details). Once the most potentially relevant predictors were selected, we evaluated if those predictors were significant in Step 2. Finally, Step 3 was addressed by evaluating the likelihood that nonsignificant variables (variables excluded from the reported models) were excluded due to masking effects or because they are simply nonsignificant predictors.

In both the first and second steps, conditional random forest and conditional regression tree techniques were employed (Strobl et al. 2009). To provide some background, classification and regression trees (CART), specifically conditional regression trees in our case, are non‐parametric techniques that involve recursive partitioning—an algorithmic process of defining the best statistically significant splits or subsets of the data using frequentist statistics (Berk 2020; LeDell 2018; Strobl et al. 2009). CART models can handle a large amount of regressors without overfitting (e.g., unlike generalized linear models, where adding more predictors can artificially inflate *R* ^2^ and reduce replicability across data sets) (Berk 2020; Breiman 2001; LeDell 2018), they capture interactions without specifying them (e.g., demonstrating different variable combinations leading to similar dependent variable scores), and they are not limited by assumptions about dependent variable distributions or independency among the regressors (i.e., no problems derived from multicollinearity) (Strobl et al. 2009). Additionally, these CART models, or trees, can be summed up to build a random forest. A random forest is a model built by many trees (usually 1000), where each tree uses only a subset of the total regressors to predict the dependent variable, and the predictors are randomly assigned to each tree in the forest.

During the first step, we used a conditional random forest for variable screening. The main purpose of this step was to obtain the variable importance of each regressor to then rank them. Variable importance is a measure of the reduction in the model's error resulting from the inclusion of a variable. As such, higher variable importance indicates better predictive value. However, given that error is not provided as a standardized unit in these models, variable importance should be interpreted as relative to other variables in the same model.

In the model we constructed, general relationship satisfaction (CSI Total) and sexual satisfaction (NSSS Total) were set as outcome variables and standardized to Z‐scores. The 56 regressors included subscale and total scores for the measures of intrapersonal and interpersonal mindfulness, social and couple‐related factors, and emotional wellbeing mentioned above, as well as relevant demographic items (i.e., age, gender, sexual orientation, relationship length—months, relationship status, and meditation experience). Given the random nature of random forests, we performed 1000 iterations of naïve bootstrap to increase external validity and estimate confidence intervals for the variable importances. Additionally, the number of regressors sampled at each split in the trees was defined as the rounded square root of the total number of regressors (mtry = 7) following recommendations to prevent overfitting (Hastie et al. 2009). Upon visual inspection of the variable importance ranking, we selected the potentially relevant predictors for the next step. Importantly, a high variable importance does not necessarily indicate that the regressor is a significant predictor, which leads to our second step of confirming predictor significance.

During the second step, our aim was to establish if the potentially relevant predictors selected in Step 1 are in fact significant predictors. In doing so, we fitted a conditional CART model. We selected these models for the reasons mentioned above (see beginning of Analysis Strategy) and because random forests are highly opaque, making interpretation difficult even with only significant predictors. Compared to random forests, CART models are easily and intuitively interpretable. As such, we entered the variables selected in Step 1 to evaluate which of them remain in the model, therefore distinguishing them as significant predictors (*p* < 0.05). However, CART models can present masking effects resulting in the exclusion of significant predictors, which leads to our third step of assessing masking effects.

In contrast with traditional approaches such as multiple linear regression which can be impacted by multicollinearity issues, random forests and regression trees do not present the same issues. Instead, these models can account for multicollinearity (i.e., strong correlations among multiple predictor variables; Meyers et al. 2017) through a process of masking (i.e., only one of two or more strongly correlated predictor variables appears in the tree; Doyle 1973). While multicollinearity is more complex than masking and can eventually lead to masking effects, issues persist when masking is absent because variable selection does not remove the colinear variable. Consequently, multicollinearity can produce inflated standard errors and poor coefficient estimations (including potential sign reversions) which in turn can lead to instability (small sample changes produce qualitatively different results) and a lack of external validity of the results (Kim 2019; Vatcheva et al. 2016). Additionally, without yet accounting for the shared variance, it is not possible to properly interpret the contribution of each regressor. Alternatively, masking effects deal with these shortcomings by selecting one of the collinear variables. Collinear variables share variance, and therefore, information. As a result, both can be used in the same split up to some point in a CART model. Then, the algorithm selects the variable that produces the best‐fitting split and drops the other variable not due to a lack of significance, but rather because it is redundant (i.e., correlates with an already selected variable). As such, we may misinterpret dropped variables as nonsignificant when performing Step 2. To assess this limitation in Step 3, we explored the Pearson correlation matrices for the variables selected during Step 1. By assessing the strength of the regressors’ correlations and iteratively removing one of two strongly correlated variables from the CART models constructed during Step 2, we were able to explore up to which point a variable was excluded from the model due to redundancy (i.e., masking) versus insignificance.

R statistical software, along with packages such as party and jtools, was used to process and analyze the data for all analyses (Hothorn et al. 2025; Long 2024; R Core Team 2023). The maximum available data was used for each analysis, and participants with any missing values in the measures of interest were omitted. Accordingly, demographic and internal consistency analyses included the maximum available datapoints of the full sample (*N* = 356), whereas variable importance analyses included only participants with complete data on all variables of interest (*N* = 246). Missingness occurred in entire survey sections rather than single items, indicating that it was not completely random and could compromise the reliability of imputation estimates (Berrevoets et al. 2023). In this case, since missingness also represented greater than 20%–25% of our sample (approximately 30%), we decided not to perform data imputation procedures due to the risk of introducing significant bias and reducing external validity (Alwateer et al. 2024; Kowsar et al. 2024; Liu and Sriutaisuk 2021; Nguyen 2020). Although there are currently no definitive guidelines for the sample size required in machine‐learning approaches, it is suggested that a 1:2 predictor‐to‐observation ratio does not introduce major issues with accuracy or overfitting (Vabalas et al. 2019). Given that each of the 1000 models composing the random forest presented seven regressors in Step 1, and each of the two CART models presented six regressors in Step 2, the proportion of predictors to observations was deemed sufficient even with a sample size of 246.

## 3 Results

### 3.1 Random Forests Exploring Variable Importance for Relationship and Sexual Satisfaction

**Figure 1:**
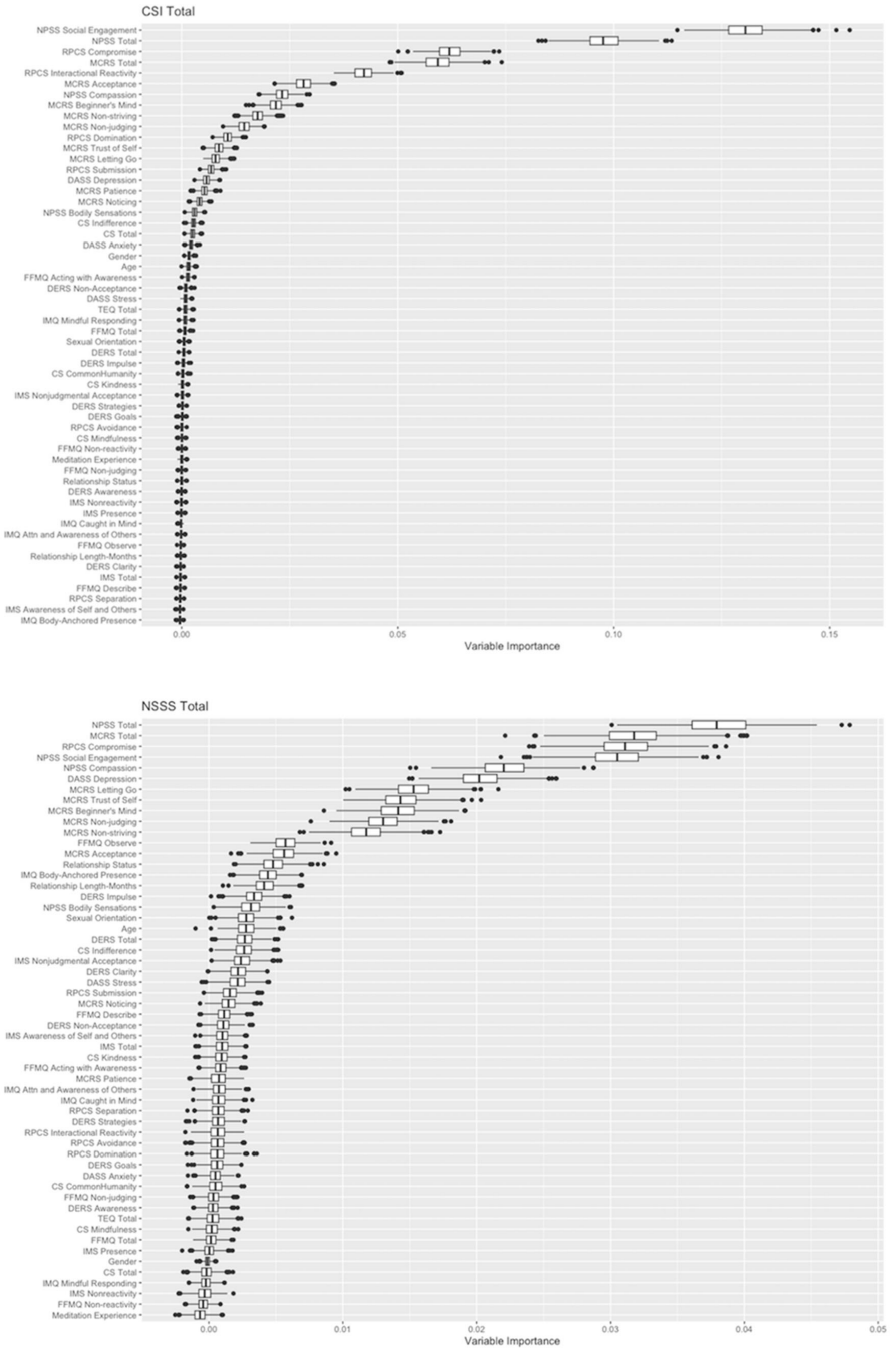
Variable importance plot for Relationship (CSI Total) and Sexual Satisfaction (NSSS Total). *Note:* The plots show the importance of each variable in the random forest model. Boxplots described the 1000 bootstrap variable importance values, with boxplot whiskers covering 95%CI. Outcome values were standardized to Z‐score. Higher values indicate greater importance in predicting Relationship (CSI Total) and Sexual Satisfaction (NSSS Total). CI, confidence interval; CSI, Couples Satisfaction Index; NSSS, new sexual satisfaction scale.

Following Step 1 (variable screening), the variable importance plots depicted the most to least important variables associated with relationship satisfaction and sexual satisfaction (see Figure 1). Several variables appeared to hold greater importance above many others which paled in comparison. When considering relationship satisfaction, the subscale and total scores for NPSS, RPCS, and MCRS were highly important and ranked among the top six variables. The plot for CSI Total illustrates how context‐specific interpersonal mindfulness and couple‐related factors outranked demographic variables and other measures of mindfulness (i.e., FFMQ, IMQ, and IMS), social factors (i.e., CS and TEQ) and those of emotional wellbeing (i.e., DASS and DERS). When considering sexual satisfaction, the subscale and total scores for NPSS, RPCS, and MCRS similarly emerged as highly important and ranked among the top six variables. However, the depression symptoms assessed with DASS also appeared to play an important role in sexual satisfaction, more so than in relationship satisfaction. The plot for NSSS Total again illustrates how context‐specific interpersonal mindfulness and couple‐related factors outranked other variables, except for depression symptoms which performed better in this context. At this stage, the top six variables for both relationship and sexual satisfaction were selected as potentially relevant variables that would undergo further analysis, as variable importance rapidly declined after these six.

#### 3.1.1 Evaluating Top Six Variables for Relationship Satisfaction

**Figure 2:**
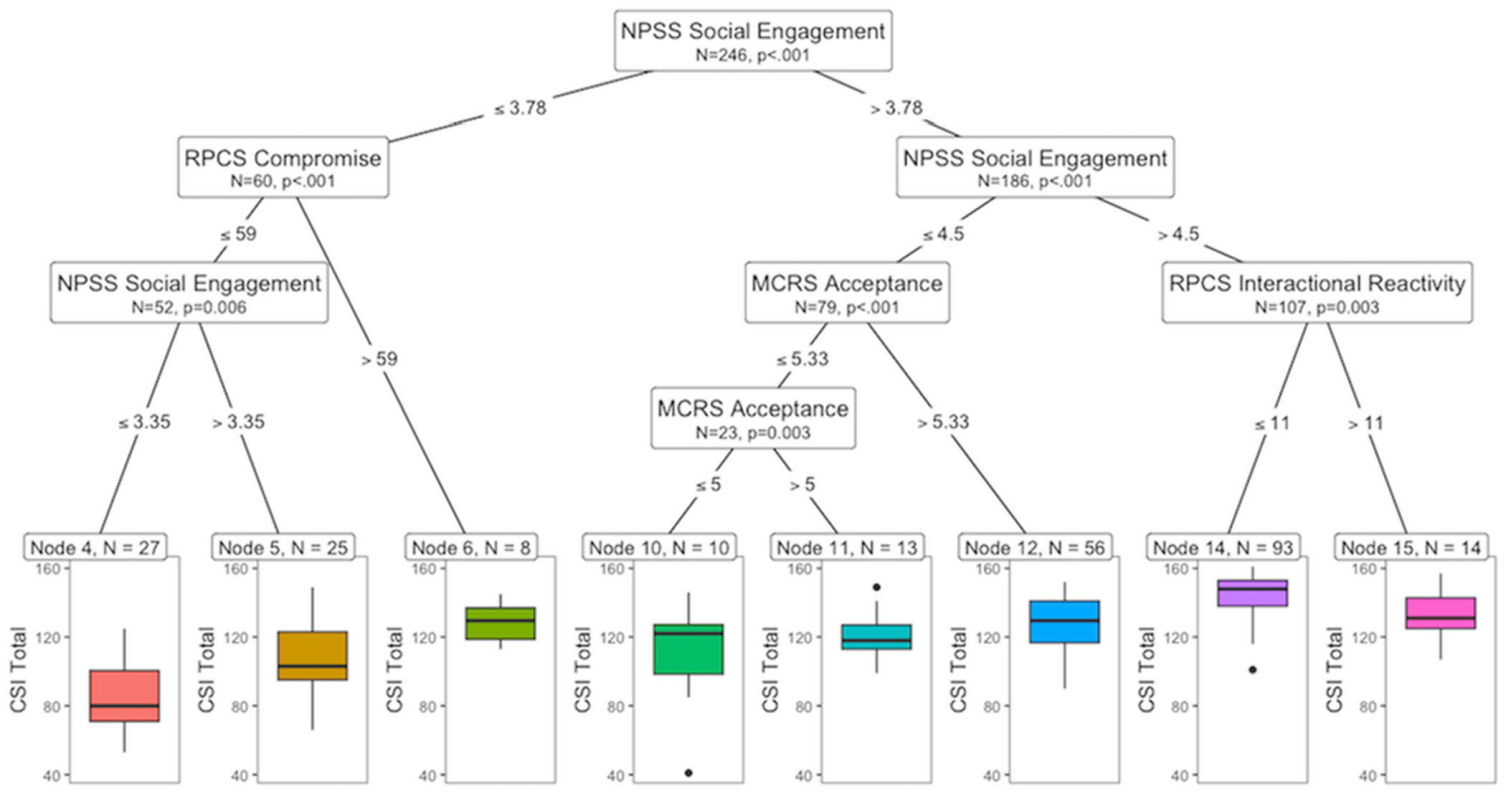
Regression tree for relationship satisfaction (CSI Total). *Note:* The regression tree presents profiles associated with the lowest to highest levels of Couples Satisfaction Index (CSI Total). Each node represents a splitting point based on statistical significance (*p* < 0.05). Terminal nodes indicate the predicted value of CSI Total and the proportion of observations. [Color figure can be viewed at wileyonlinelibrary.com]

As outlined in Step 2 (confirmation of predictor significance), to assess which profiles were associated with the lowest to highest levels of relationship satisfaction, we ran a regression tree further evaluating the top six variables for CSI Total. In this case, the top six variables were NPSS Social Engagement, NPSS Total, RPCS Compromise, MCRS Total, RPCS Interactional Reactivity, and MCRS Acceptance. The root node defining the primary and best split in the data was NPSS Social Engagement with values > or ≤ 3.78 (refer to Figure 2, Node 1). At the extremes, it appears that lower NPSS Social Engagement and RPCS Compromise contribute to the lowest levels of CSI Total (Figure 2, Node 4), whereas higher NPSS Social Engagement and lower RPCS Interactional Reactivity contribute to the highest levels of CSI Total (Figure 2, Node 14). Other conditions displayed within the tree demonstrate how higher MCRS Acceptance and RPCS Compromise may compensate for deficits in NPSS Social Engagement (Figure 2, Nodes 6, 10, 11, and 12).

**Figure 3:**
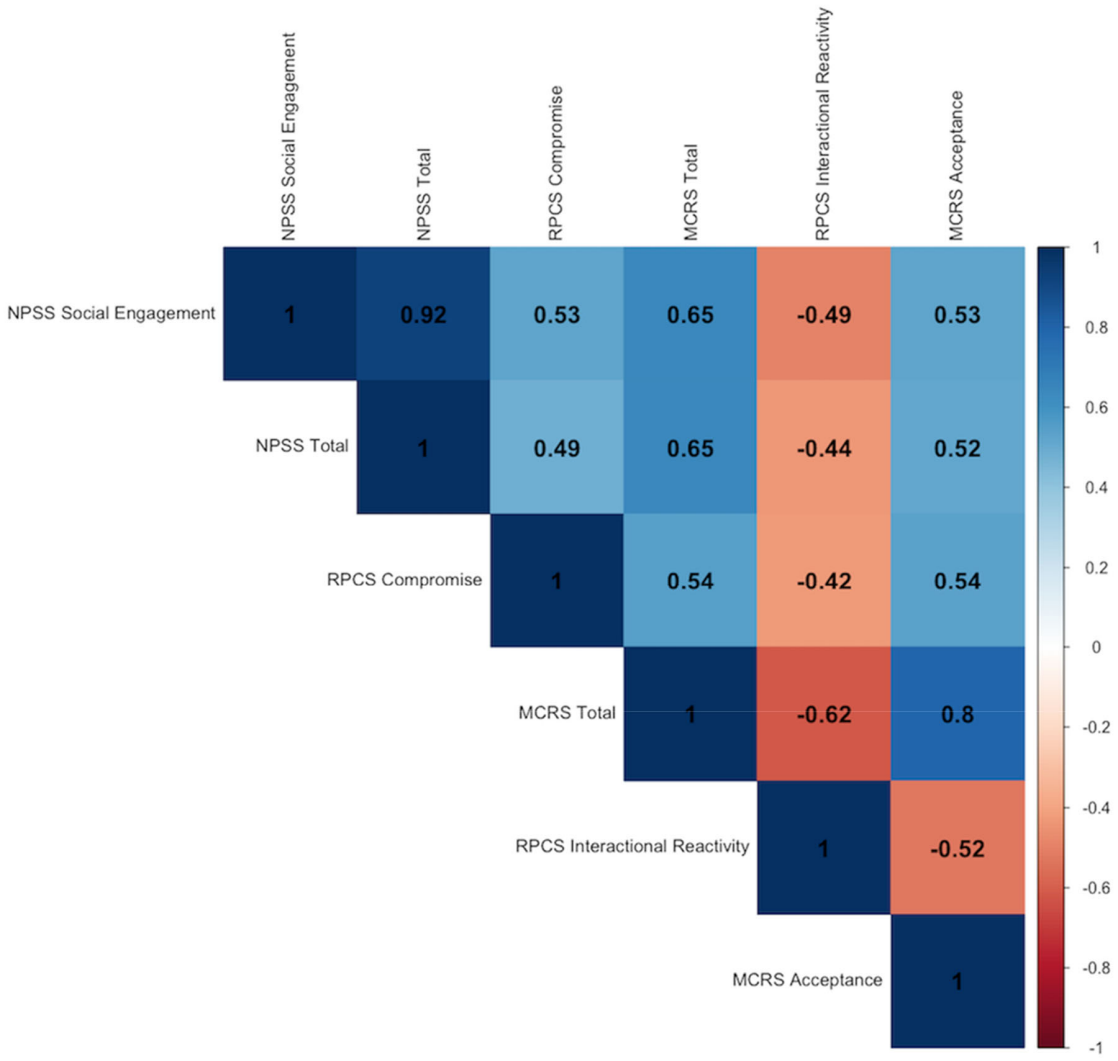
Pearson correlation matrix of top six variables for relationship satisfaction. *Note:* The correlation matrix presents significant associations (*p* < 0.05) between the top six variables for relationship satisfaction, including Neuroception of Psychological Safety Scale (i.e., NPSS Social Engagement, Total), Romantic Partner Conflict Scale (i.e., RPCS Compromise, Interactional Reactivity), and Mindfulness in Couple Relationships Scale (i.e., MCRS Total, Acceptance). Pearson's *r* is presented in a scale of colors (depicted to the right) as well as a value in each cell. [Color figure can be viewed at wileyonlinelibrary.com]

Proceeding with Step 3 (assessment of masking effects), all the top six variables initially entered in the regression trees presented significant correlations (*p* < 0.05), and the results are presented in Figure 3. Most of the correlations were high (Pearson's *r* > 0.5 or *r* < −0.5) except for some of the associations between RPCS Compromise, RPCS Interactional Reactivity, NPSS Social Engagement, and NPSS Total (Pearson's *r* ranging from −0.49 to 0.49). Together, the results provide an explanation for how the missing variables in the regression tree (i.e., NPSS Total, MCRS Total) were masked as irrelevant due to their high association with each other and with other variables (i.e., NPSS Social Engagement, MCRS Acceptance), despite being significant regressors when the latter variables were removed.

#### 3.1.2 Evaluating Top Six Variables for Sexual Satisfaction

**Figure 4:**
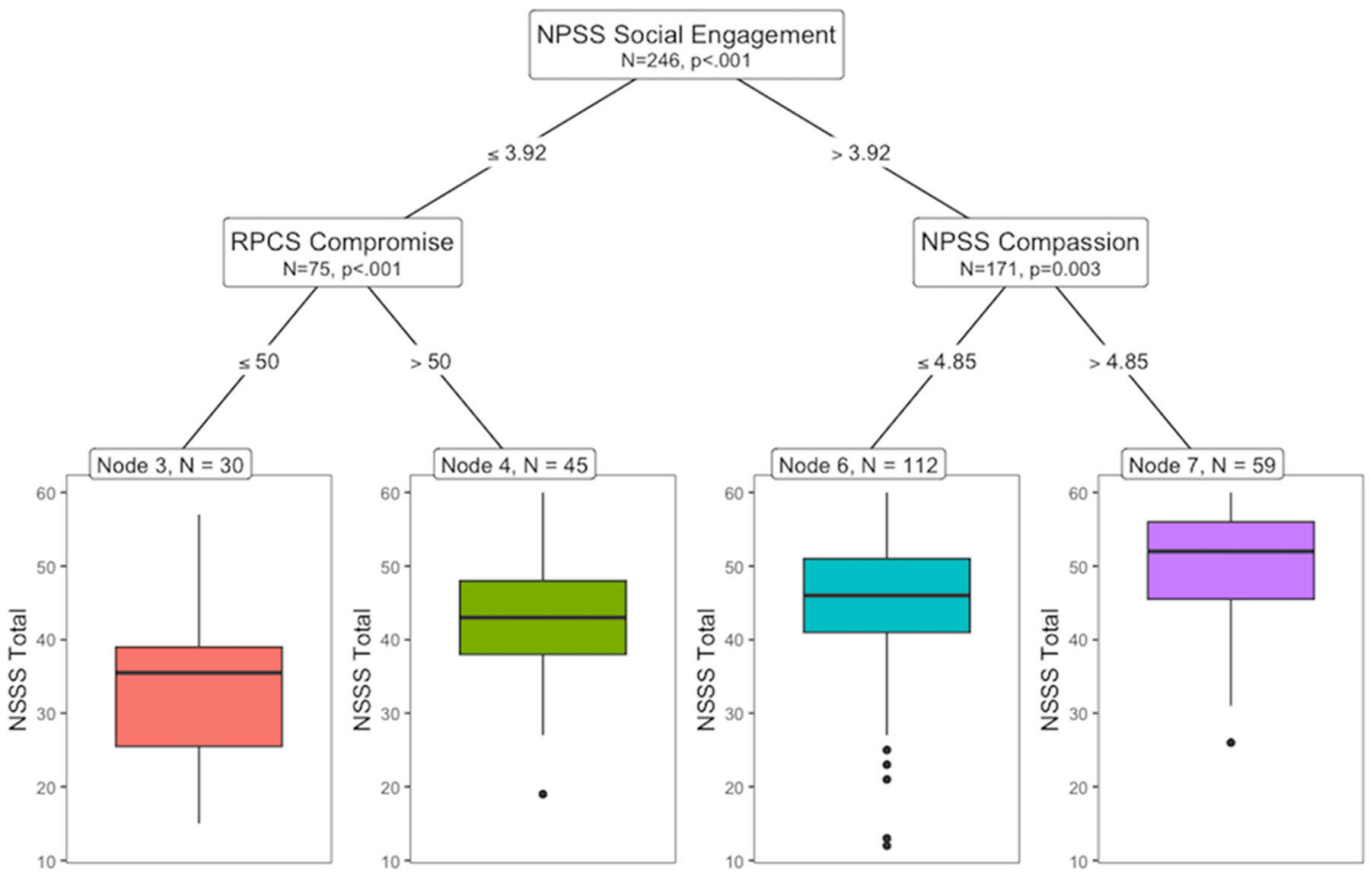
Regression tree for sexual satisfaction (NSSS Total). *Note:* The regression tree presents profiles associated with the lowest to highest levels of New Sexual Satisfaction Scale ‐ Short (NSSS Total). Each node represents a splitting point based on statistical significance (*p* < 0.05). Terminal nodes indicate the predicted value of NSSS Total and the proportion of observations. [Color figure can be viewed at wileyonlinelibrary.com]

As outlined in Step 2, to assess which profiles were associated with the lowest to highest levels of sexual satisfaction, we also ran a regression tree further evaluating the top six variables for NSSS Total. In this case, the top six variables were NPSS Total, MCRS Total, RPCS Compromise, NPSS Social Engagement, NPSS Compassion, and DASS Depression. The root node defining the primary and best split in the data was NPSS Social Engagement with values > or ≤ 3.92 (refer to Figure 4, Node 1). At the extremes, it appears that lower NPSS Social Engagement and RPCS Compromise contribute to the lowest levels of NSSS Total (Figure 4, Node 3), whereas higher NPSS Social Engagement and NPSS Compassion contribute to the highest levels of NSSS Total (Figure 4, Node 7). Other conditions displayed within the tree demonstrate how higher RPCS Compromise may compensate for deficits in NPSS Social Engagement (Figure 4, Node 4).

**Figure 5:**
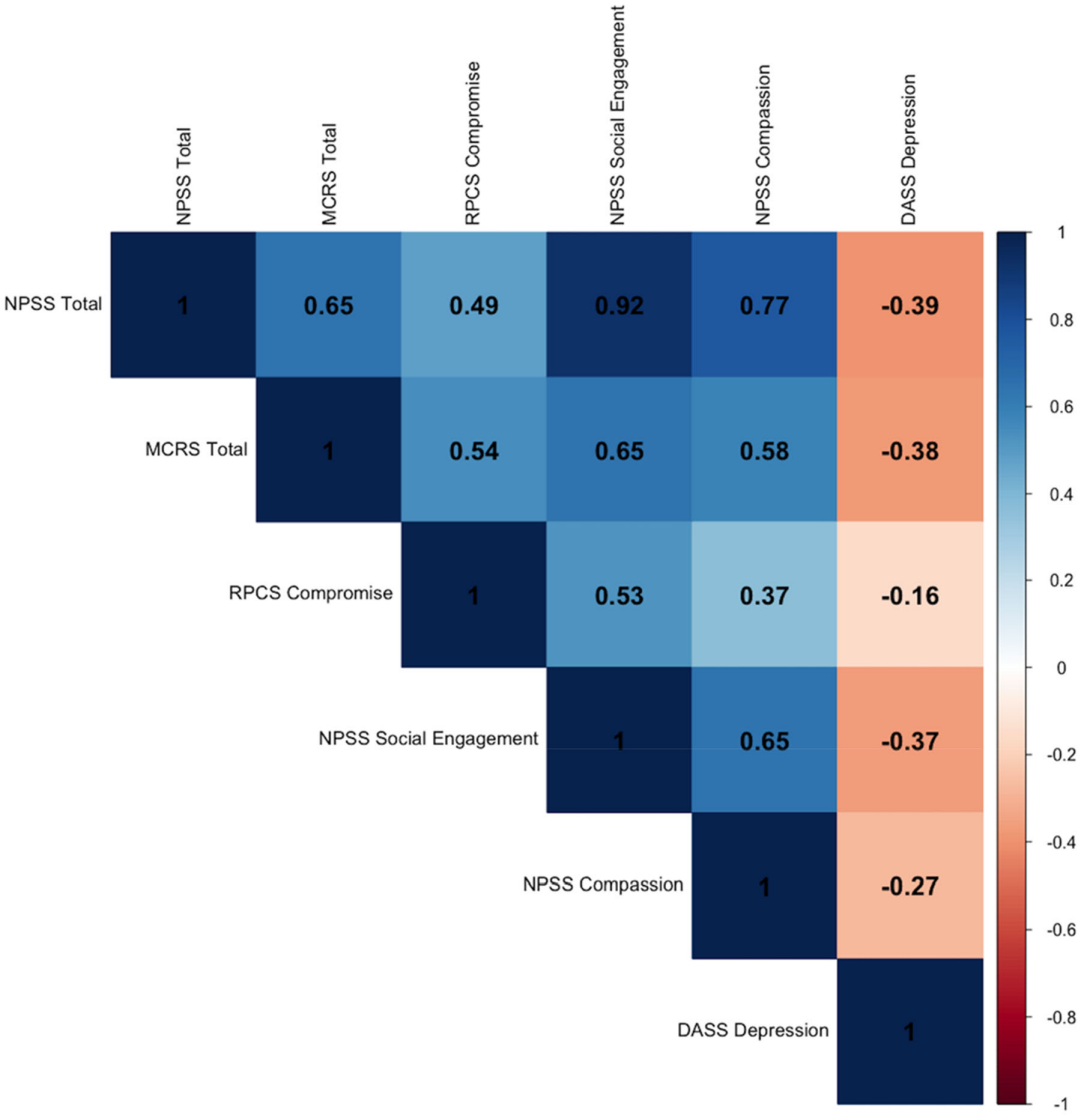
Pearson correlation matrix of top six variables for sexual satisfaction. *Note:* The correlation matrix presents significant associations (*p* < 0.05) between the top six variables for sexual satisfaction, including Neuroception of Psychological Safety Scale (i.e., NPSS Total, Social Engagement, Compassion), Romantic Partner Conflict Scale (i.e., RPCS Compromise), Mindfulness in Couple Relationships Scale (i.e., MCRS Total), and Depression Anxiety Stress Scales (i.e., DASS Depression). Pearson's *r* is presented in a scale of colors (depicted to the right) as well as a value in each cell. [Color figure can be viewed at wileyonlinelibrary.com]

Proceeding with Step 3, all the top six variables initially entered in the regression trees presented significant correlations (*p* < 0.05). As presented in Figure 5, most of the correlations were medium to high (Pearson's *r* > 0.35 or *r* < −0.35) except for some of those between DASS Depression and other variables which were low (Pearson's *r* ranging from −0.16 to −0.27). Again, the results highlight how the missing variables in the regression tree (i.e., NPSS Total, MCRS Total) were masked as irrelevant due to their high association with each other and with other variables (i.e., NPSS Social Engagement, NPSS Compassion), despite being significant regressors when the latter variables were removed. In contrast, the significance of DASS Depression only appeared to emerge after all NPSS variables were removed and its interactions with MCRS Total were present in the tree.

## 4 Discussion

The primary objective of the current study was to determine the variable importance of interpersonal mindfulness and psychological safety for relationship and sexual satisfaction, as well as explore the importance of several other demographic, mindfulness, social and couple‐related, and emotional wellbeing variables in this analysis. Recruitment of a sexually and racially diverse sample, and the use of machine‐learning techniques, provided novelty in this preliminary analysis of variable importance. Although previous research had employed machine‐learning techniques to assess variable importance for relationship and sexual satisfaction (Joel et al. 2020; Vowels et al. 2022), to the best of our knowledge, the current study is the first to include context‐specific interpersonal mindfulness as an independent variable in this type of analysis while also distinguishing between relationship and sexual satisfaction as dependent variables given that both are commonly assessed as outcomes in the evaluation of clinical interventions (McClelland 2011; Wood et al. 2005).

Findings from Step 1 illustrate that context‐specific interpersonal mindfulness (i.e., MCRS), couple‐related factors (i.e., NPSS and RPCS), and depression symptoms (i.e., DASS) appear to be of top importance for relationship and sexual satisfaction. More specifically, during Step 2, we found that greater relationship satisfaction appears to be associated with higher levels of psychological safety (social engagement), mindfulness in couple relationships (acceptance), and constructive conflict strategies (compromise), as well as lower levels of destructive conflict strategies (interactional reactivity). Whereas greater sexual satisfaction appears to be associated with higher levels of psychological safety (social engagement and compassion) and constructive conflict strategies (compromise). The findings suggest that in addition to interpersonal mindfulness and psychological safety, conflict strategies are clearly relevant and seem to interact in different ways, as evidenced by the recurrence of subscales throughout multiple branches of the regression trees.

Additionally, as per Step 3, correlations among the top six variables for each type of satisfaction provided insight into the masking effects of certain variables entered in the regression trees. For example, total scores for psychological safety and mindfulness in couple relationships appeared to be significant regressors of both relationship and sexual satisfaction when other strongly correlated regressors were iteratively removed from the respective tree. Moreover, depression symptoms appeared to be a significant regressor of sexual satisfaction when all psychological safety variables were removed, suggesting that their impact may be linked to interactions with the total score for mindfulness in couple relationships. Interestingly, including both total and subscale scores in these analyses provided valuable insights into the performance of the general constructs compared to their specific components, highlighting which aspects were most influential for relationship and sexual satisfaction.

Our findings align well with growing research in this area. As previously mentioned, researchers employing machine‐learning techniques suggest that relationship‐specific variables tend to be more reliable predictors of relationship and sexual satisfaction in comparison to objective relationship variables and individual difference variables (Joel et al. 2020; Vowels et al. 2022). Moreover, trait mindfulness has repeatedly been linked with satisfaction and researchers have begun to operationalize interpersonal mindfulness for more context‐specific analyses (e.g., Atkinson 2013; Daks et al. 2021; Kimmes et al. 2018; Kozlowski 2013; McGill et al. 2016, 2022; Quinn‐Nilas 2020b). Alongside the theoretical underpinnings of Porges' PVT (1995, 1998, 2004, 2011, 2017) and suggestions to integrate mindfulness and compassion practices in couples therapy (e.g., Bradford and Johnson 2023; Fox 2024; Gilbert 2020; Ryland et al. 2022), existing literature provided a basis for understanding how interpersonal mindfulness and psychological safety would be interrelated in the context of romantic relationships, and highly associated with conflict strategies.

In summary, the couple‐related variables (e.g., mindfulness in couple relationships, psychological safety, and conflict strategies) in our study appeared to outrank objective relationship variables and individual difference variables (i.e., demographics, other forms of mindfulness, compassion, empathy, and emotional wellbeing), with the exception of depression symptoms in relation to sexual satisfaction. It is possible that depression symptoms are particularly relevant for sexual satisfaction as they capture loss of interest or pleasure in activities and have been linked with sexual dissatisfaction/dysfunction, which could also be a potential side effect of antidepressants (Sánchez‐Fuentes et al. 2014; Henry and Crawford 2005). Overall, the current findings confirmed the hypothesis that interpersonal mindfulness and psychological safety are of greater importance to satisfaction compared to intrapersonal mindfulness and the other variables of interest, and further underscored that conflict strategies play a key role. The findings provide a foundation for future investigations into the links between interpersonal mindfulness, psychological safety, and conflict strategies in romantic relationships.

### 4.1 Limitations

The current study has several limitations. First, the sample consisted of predominantly women (79.49%), heterosexual (65.73%), White (74.16%), and only Canadian participants. As the field of relationship science continues to suffer from a lack of diversity (Williamson et al. 2022), efforts were made to recruit a sexually and racially diverse sample. Given that 4.4% of the Canadian population (aged 15 + ) identifies as Two‐Spirit, lesbian, gay, bisexual, transgender, queer, or other terms related to gender or sexual diversity, the representation of sexual and gender minorities in this study is at least proportional to the population prevalence (Statistics Canada 2024). Nonetheless, a limitation associated with proportional representation is the possibility of under‐powered generalizability (Williamson et al. 2022). Second, the omission of participants with missing values from the variable importance analyses reduced the number of observations to 246. However, given the risks associated with imputing 30% of observations, coupled with the recommended ratio of 1:2 predictors‐to‐observations being met, the sample size was deemed sufficient to maintain accuracy and avoid overfitting in these analyses (Alwateer et al. 2024; Kowsar et al. 2024; Liu and Sriutaisuk 2021; Nguyen 2020; Vabalas et al. 2019).

The design of the current study presents additional limitations in the scope and applicability of results. Since the results are based on data collected at one timepoint and from individuals rather than both partners in a couple, the findings were unable to capture aspects of causality or possible dyadic effects. Researchers who previously used machine learning to assess predictors of satisfaction found that partner effects did not explain much of the additional variance over individual effects (Joel et al. 2020; Vowels et al. 2022). Nonetheless, emergent literature on mindfulness in couples highlights both a growing interest in the topic and inconsistencies in its individual and dyadic influences on relationship functioning (e.g., Karremans et al. 2017; Khaddouma et al. 2017; Kimmes et al. 2025; Stanton et al. 2021). Without further research using a longitudinal and dyadic approach to examine the relative importance of these variables, identifying key treatment targets and determining the value of involving one or both partners in mindfulness‐ and compassion‐based interventions remains challenging.

Finally, the measures administered at the time of data collection did not include other known predictors of relationship and sexual satisfaction, which could potentially change the relative rankings of importance if included in the random forest model. Notably, researchers have previously considered variables such as perceived commitment, desire, appreciation, responsiveness, sexual communication, and sexual mindfulness (Adair et al. 2018; Joel et al. 2020; Khalifian and Barry 2021; Leavitt et al. 2019; Smedley et al. 2021; Vowels et al. 2022). While the omission of additional questionnaires was intended to prevent survey fatigue, it is worth noting that the exclusive use of self‐report measures has its own limitations (e.g., sentiment override, shared method variance). Specifically, there is a growing emphasis on the need to address sentiment override (wherein spouses’ global sentiments toward their partner or marriage can *positively* or *negatively* override their perception of a *neutral* interaction, or in the case of research, a self‐report measure), control for shared method variance (wherein reliance on a single method such as self‐report measures can inflate or deflate associations among variables), and ultimately enhance the validity and objectivity of research measures and results in relationship science (Hawkins et al. 2002; Orth 2013; Weiss 1980).

### 4.2 Future Directions

Future research could address the above limitations and strengthen methodology beyond the scope of the current study. Regarding diversity and inclusion, researchers hold a responsibility to increasingly and respectfully involve underrepresented groups in relationship science (Williamson et al. 2022). At the time of writing this paper, the first author is collecting data for a second time point which would allow us to further assess mediation effects between mindfulness, psychological safety, and conflict in the prediction of satisfaction. However, future studies would benefit from additional timepoints or the use of ecological momentary assessment to examine ongoing fluctuations in interpersonal mindfulness and psychological safety in couples (e.g., daily diary methodology; Gazder and Stanton 2020; Morin et al. 2024). Together, these considerations would provide greater insight into the generalizability of predictors of relationship and sexual satisfaction over time, and the potential of learnable skills (e.g., relaxation or mindfulness exercises, polyvagal cues of safety) that can be leveraged in therapy and in life to support social engagement, compassion, and coregulation in romantic relationships.

Additionally, future studies could involve both partners in a couple and further investigate dyadic effects using actor‐partner interdependence models (APIM; Kenny et al. 2006), multilevel modeling, and machine‐learning techniques with larger cross‐study samples (e.g., Gazder and Stanton 2020; Morin et al. 2024; Joel et al. 2020). To address sentiment override and shared method variance in relationship science, it will also be crucial to validate novel measures, such as the NPSS, in couples and employ multimethod approaches to assess relationship‐specific variables. By collecting actor and partner reports, integrating observational techniques, and assessing neurophysiological indicators of vagal activity and psychological safety (e.g., heart rate variability; Berntson et al. 1993; Bradford and Johnson 2023; Petrocchi and Cheli 2019), a more comprehensive and complex understanding of the role of interpersonal mindfulness and psychological safety in romantic relationships can be achieved.

### 4.3 Clinical Implications and Conclusion

The findings present several clinical implications for the improvement of relationship and sexual satisfaction in couples therapy. Considering the role of depression in sexual satisfaction specifically, researchers have suggested that individual and relational meditation practices may be helpful adjuncts to couples therapy and found that mindfulness‐based interventions can improve depressive symptoms (May et al. 2020; Winter et al. 2021). To address broader distress in couples therapy, the current study further supports existing recommendations for providing psychoeducation on polyvagal theory and integrating mindfulness‐ and compassion‐based strategies that help clients down‐regulate, cultivate connection, and resolve conflict more constructively (Bradford and Johnson 2023; Fox 2024; Lebow and Snyder 2022; Ryland et al. 2022; Siegel 2014; Winter et al. 2021). In this context, therapists are expected to play a crucial role by modeling therapeutic presence and identifying compassionate and co‐regulating behaviors between partners over repeated interactions (Geller and Porges 2014; Porges and Dana 2018; Ryland et al. 2022). Taking it a step further, a more comprehensive clinical intervention for enhancing interpersonal mindfulness and psychological safety—aligned with the Embodied and Embedded Mindfulness and Compassion Framework—could focus on cultivating mindfulness and compassion synergistically, both toward oneself and others, within broader intrapersonal, interpersonal, and environmental contexts (Khoury 2018, 2019; Khoury et al. 2017, 2025). Continued research is needed to assess these implications in practice and to inform the refinement of interpersonal mindfulness‐ and polyvagal‐based interventions for diverse individuals and couples in romantic relationships. This line of research will be critical to understanding how to promote safety and stability in romantic relationships and to equip therapists with the tools to manage the reactivity of their own and their clients’ nervous systems in sessions.

## Ethics Statement

The study reported in this manuscript received ethics approval at the host university (i.e., McGill University REB #23‐08‐068). Written informed consent was obtained from all individual participants included in the study.

## Supporting information

Descriptive Results of Instruments Administered.

## Data Availability

The data that support the findings of the current research are not publicly available as per restrictions posed by the ethics certificate from the host university (i.e., McGill University). The code for statistical analyses is available upon request.

## References

[jmft70026-bib-0001] Adair, K. C. , A. J. Boulton , and S. B. Algoe . 2018. “The Effect of Mindfulness on Relationship Satisfaction via Perceived Responsiveness: Findings From a Dyadic Study of Heterosexual Romantic Partners.” Mindfulness 9: 597–609. 10.1007/s12671-017-0801-3.

[jmft70026-bib-0002] Agnew, C. R. , and S. C. South . 2014. Interpersonal relationships and health: Social and Clinical Psychological Mechanisms. Oxford University Press. 10.1093/acprof:oso/9780199936632.001.0001.

[jmft70026-bib-0003] Alwateer, M. , E.‐S. Atlam , M. M. A. El‐Raouf , O. A. Ghoneim , and I. Gad . 2024. “Missing Data Imputation: A Comprehensive Review.” Journal of Computer and Communications 12: 53–75. 10.4236/jcc.2024.1211004.

[jmft70026-bib-0004] Atkinson, B. J. 2013. “Mindfulness Training and the Cultivation of Secure, Satisfying Couple Relationships.” Couple and Family Psychology: Research and Practice 2, no. 2: 73–94. 10.1037/cfp0000002.

[jmft70026-bib-0005] Baer, R. A. , J. Carmody , and M. Hunsinger . 2012. “Weekly Change in Mindfulness and Perceived Stress on a Mindfulness‐Based Stress Reduction Program.” Journal of Clinical Psychology 68, no. 7: 755–765. 10.1002/jclp.21865. 22623334

[jmft70026-bib-0006] Baer, R. A. , G. T. Smith , J. Hopkins , J. Krietemeyer , and L. Toney . 2006. “Using Self‐Report Assessment Methods to Explore Facets of Mindfulness.” Assessment 13, no. 1: 27–45. 10.1177/1073191105283504. 16443717

[jmft70026-bib-0007] Berk, R. A. 2020. “Random Forests.” In Statistical learning from a regression perspective, 233–295. Springer. 10.1007/978-3-030-40189-4_5.

[jmft70026-bib-0008] Berntson, G. G. , J. T. Cacioppo , and K. S. Quigley . 1993. “Respiratory Sinus Arrhythmia: Autonomic Origins, Physiological Mechanisms, and Psychophysiological Implications.” Psychophysiology 30, no. 2: 183–196. 10.1111/j.1469-8986.1993.tb01731.x. 8434081

[jmft70026-bib-0009] Berrevoets, J. , F. Imrie , T. Kyono , J. Jordon , and M. van der Schaar . 2023. “To Impute or Not to Impute? Missing Data in Treatment Effect Estimation.” Proceedings of the 26th International Conference on Artificial Intelligence and Statistics, in Proceedings of Machine Learning Research, Spain 206: 3568–3590. https://proceedings.mlr.press/v206/berrevoets23a.html.

[jmft70026-bib-0010] Bodhi, B. 2011. “What Does Mindfulness Really Mean? A Canonical Perspective.” Contemporary Buddhism 12, no. 1: 19–39. 10.1080/14639947.2011.564813.

[jmft70026-bib-0011] Bradford, A. B. , and L. N. Johnson . 2023. “What to Expect Physiologically When You'Re Expecting Couples in Therapy: A Changing Hearts and Minds in Relationships (Champs) Descriptive Study.” Journal of Marital and Family Therapy 49, no. 1: 222–241. 10.1111/jmft.12619. 36378837

[jmft70026-bib-0012] Breiman, L. 2001. “Random Forests.” Machine Learning 45, no. 1: 5–32. 10.1023/A:1010933404324.

[jmft70026-bib-0013] Brouillard, P. , A. Stulhofer , and V. Busko . 2020. “Pleasure, Satisfaction, and Orgasm: The New Sexual Satisfaction Scale and Its Short Form.” In Handbook of sexuality‐related measures (4th ed, edited by R. R. Milhausen , J. K. Sakulak , T. D. Fisher , C. M. Davis , and W. L. Yarber , 495–497. Routledge. 10.4324/9781315183169.

[jmft70026-bib-0014] Brown, K. W. , and R. M. Ryan . 2003. “The Benefits of Being Present: Mindfulness and Its Role in Psychological Well‐Being.” Journal of Personality and Social Psychology 84, no. 4: 822–848. 10.1037/0022-3514.84.4.822. 12703651

[jmft70026-bib-0015] Byers, E. S. 2005. “Relationship Satisfaction and Sexual Satisfaction: A Longitudinal Study of Individuals in Long‐Term Relationships.” Journal of Sex Research 42, no. 2: 113–118. 10.1080/00224490509552264. 16123841

[jmft70026-bib-0016] Carr, D. , and K. W. Springer . 2010. “Advances in Families and Health Research in the 21st Century.” Journal of Marriage and Family 72, no. 3: 743–761.

[jmft70026-bib-0017] Cozolino, L. 2006. The Neuroscience of Human Relationships: Attachment and the Developing Social Brain. W. W. Norton & Co.

[jmft70026-bib-0018] Daks, J. S. , R. D. Rogge , and F. D. Fincham . 2021. “Distinguishing the Correlates of Being Mindfully vs. Mindlessly Coupled: Development and Validation of the Attentive Awareness in Relationships Scale (AAIRS).” Mindfulness 12, no. 6: 1361–1376. 10.1007/s12671-021-01604-w.

[jmft70026-bib-0019] Dana, D. 2021. “Anchored: How to Befriend Your Nervous System Using Polyvagal Theory.” Sounds True.

[jmft70026-bib-0020] Davison, S. L. , R. J. Bell , M. LaChina , S. L. Holden , and S. R. Davis . 2009. “The Relationship Between Self‐Reported Sexual Satisfaction and General Well‐Being in Women.” Journal of Sexual Medicine 6, no. 10: 2690–2697. 10.1111/j.1743-6109.2009.01406.x. 19817981

[jmft70026-bib-0021] Don, B. P. , and S. B. Algoe . 2020. “Impermanence in Relationships: Trait Mindfulness Attenuates the Negative Personal Consequences of Everyday Dips in Relationship Satisfaction.” Journal of Social and Personal Relationships 37, no. 8–9: 2419–2437.

[jmft70026-bib-0022] Doyle, P. 1973. “The Use of Automatic Interaction Detector and Similar Search Procedures.” Journal of the Operational Research Society 24, no. 3: 465–467. 10.1057/jors.1973.81.

[jmft70026-bib-0023] Duncan, L. G. , J. D. Coatsworth , and M. T. Greenberg . 2009. “A Model of Mindful Parenting: Implications for Parent–Child Relationships and Prevention Research.” Clinical Child and Family Psychology Review 12, no. 3: 255–270. 10.1007/s10567-009-0046-3. 19412664 PMC2730447

[jmft70026-bib-0024] Dush, C. M. K. , and P. R. Amato . 2005. “Consequences of Relationship Status and Quality for Subjective Well‐Being.” Journal of Social and Personal Relationships 22, no. 5: 607–627. 10.1177/0265407505056438.

[jmft70026-bib-0025] Fox, D. J. 2024. “The Integration of Somatic‐Based Strategies Into Couples Therapy.” Clinical Social Work Journal 52: 79–88. 10.1007/s10615-023-00905-y.

[jmft70026-bib-0026] Funk, J. L. , and R. D. Rogge . 2007. “Testing the Ruler with Item Response Theory: Increasing Precision of Measurement for Relationship Satisfaction With the Couples Satisfaction Index.” Journal of Family Psychology 21, no. 4: 572–583. 18179329 10.1037/0893-3200.21.4.572

[jmft70026-bib-0027] Gazder, T. , and S. C. E. Stanton . 2020. “Partners' Relationship Mindfulness Promotes Better Daily Relationship Behaviours for Insecurely Attached Individuals.” International Journal of Environmental Research and Public Health 17, no. 19: 7267. 10.3390/ijerph17197267. 33027896 PMC7579090

[jmft70026-bib-0028] Geller, S. M. , and S. W. Porges . 2014. “Therapeutic Presence: Neurophysiological Mechanisms Mediating Feeling Safe In Therapeutic Relationships.” Journal of Psychotherapy Integration 24, no. 3: 178–192. 10.1037/a0037511.

[jmft70026-bib-0029] Gesell, N. , F. Niklas , S. Schmiedeler , and R. Segerer . 2020. “Mindfulness and Romantic Relationship Outcomes: The Mediating Role of Conflict Resolution Styles and Closeness.” Mindfulness 11, no. 10: 2314–2324. 10.1007/s12671-020-01449-9.

[jmft70026-bib-0030] Gilbert, P. 2017. “A Brief Outline of the Evolutionary Approach for Compassion Focused Therapy.” EC Psychology and Psychiatry 3: 218–227.

[jmft70026-bib-0031] Gilbert, P. 2020. “Compassion: From Its Evolution to a Psychotherapy.” Frontiers in Psychology 11: 586161. 10.3389/fpsyg.2020.586161. 33362650 PMC7762265

[jmft70026-bib-0032] Hastie, T. , R. Tibshirani , and J. Friedman . 2009. “Random Forests.” In The Elements of Statistical Learning. 2nd ed., 587–604. Springer. 10.1007/978-0-387-84858-7_15.

[jmft70026-bib-0033] Hawkins, M. W. , S. Carrère , and J. M. Gottman . 2002. “Marital Sentiment Override: Does It Influence Couples' Perceptions?” Journal of Marriage and Family 64, no. 1: 193–201. 10.1111/j.1741-3737.2002.00193.x.

[jmft70026-bib-0034] Henry, J. D. , and J. R. Crawford . 2005. “The Short‐Form Version of the Depression Anxiety Stress Scales (DASS‐21): Construct Validity and Normative Data in a Large Non‐Clinical Sample.” British Journal of Clinical Psychology 44, no. 2: 227–239. 16004657 10.1348/014466505X29657

[jmft70026-bib-0035] Henry, K. L. , T. P. Thornberry , and R. D. Lee . 2015. “The Protective Effects of Intimate Partner Relationships on Depressive Symptomatology Among Adult Parents Maltreated as Children.” Journal of Adolescent Health 57, no. 2: 150–156. 10.1016/j.jadohealth.2015.02.015 PMC4515193 25912653

[jmft70026-bib-0036] Holt‐Lunstad, J. , T. B. Smith , and J. B. Layton . 2010. “Social Relationships and Mortality Risk: A Meta‐Analytic Review.” PLoS Medicine 7, no. 7: e1000316. 20668659 10.1371/journal.pmed.1000316 PMC2910600

[jmft70026-bib-0037] Hothorn, T. , K. Hornik , C. Strobl , and A. Zeileis . 2025. “Party: A Laboratory for Recursive Partytioning. The Comprehensive R Archive Network.” https://cran.r-project.org/web/packages/party/index.html.

[jmft70026-bib-0038] Joel, S. , P. W. Eastwick , C. J. Allison , et al. 2020. “Machine Learning Uncovers the Most Robust Self‐Report Predictors of Relationship Quality Across 43 Longitudinal Couples Studies.” Proceedings of the National Academy of Sciences of the United States of America 117, no. 32: 19061–19071. 10.1073/pnas.1917036117. 32719123 PMC7431040

[jmft70026-bib-0039] Kabat‐Zinn, J. 2009. “Full Catastrophe Living: Using The Wisdom Of Your Body And Mind To Face Stress, Pain, And Illness.” Delta.

[jmft70026-bib-0040] Kappen, G. , J. C. Karremans , W. J. Burk , and A. Buyukcan‐Tetik . 2018. “On the Association between Mindfulness and Romantic Relationship Satisfaction: The Role of Partner Acceptance.” Mindfulness 9, no. 5: 1543–1556. 10.1007/s12671-018-0902-7. 30294389 PMC6153889

[jmft70026-bib-0041] Karney, B. R. , and T. N. Bradbury . 1995. “The Longitudinal Course of Marital Quality and Stability: A Review of Theory, Methods, and Research.” Psychological Bulletin 118, no. 1: 3–34. 10.1037/0033-2909.118.1.3. 7644604

[jmft70026-bib-0042] Karremans, J. C. , M. P. J. Schellekens , and G. Kappen . 2017. “Bridging the Sciences of Mindfulness and Romantic Relationships: A Theoretical Model and Research Agenda.” Personality and Social Psychology Review 21, no. 1: 29–49. 26563236 10.1177/1088868315615450

[jmft70026-bib-0043] Kaufman, E. A. , M. Xia , G. Fosco , M. Yaptangco , C. R. Skidmore , and S. E. Crowell . 2016. “The Difficulties in Emotion Regulation Scale ‐ Short Form (DERS‐SF): Validation and Replication in Adolescent and Adult Samples.” Journal of Psychopathology and Behavioral Assessment 38: 443–455. 10.1007/s10862-015-9529-3. PMC12788809 41522882

[jmft70026-bib-0044] Kenny, D. A. , D. A. Kashy , and W. L. Cook . 2006. Dyadic Data Analysis. Guilford Press.

[jmft70026-bib-0045] Khaddouma, A. , K. Coop Gordon , and E. B. Strand . 2017. “Mindful Mates: A Pilot Study of the Relational Effects of Mindfulness‐Based Stress Reduction on Participants and Their Partners.” Family Process 56, no. 3: 636–651. 10.1111/famp.12226. 27226408

[jmft70026-bib-0046] Khalifian, C. E. , and R. A. Barry . 2021. “The Relation Between Mindfulness and Perceived Partner Responsiveness During Couples’ Vulnerability Discussions.” Journal of Family Psychology 35: 1–10. 10.1037/fam0000666. 32338940

[jmft70026-bib-0047] Khorasani, E. , H. Farrokhi , E. Shoja , M. Moghaddam , and S. A. Kimiaei . 2021. “Mindfulness in the Context of a Romantic Relationship to Predict Relationship Satisfaction.” International Journal of Psychosocial Rehabilitation 25, no. 01: 400–413. https://www.researchgate.net/publication/352799202.

[jmft70026-bib-0048] Khoury, B. 2018. “Mindfulness: Embodied and Embedded.” Mindfulness 9, no. 4: 1037–1042. 10.1007/s12671-017-0858-z.

[jmft70026-bib-0049] Khoury, B. 2019. “Compassion: Embodied and Embedded.” Mindfulness 10, no. 11: 2363–2374. 10.1007/s12671-019-01211-w.

[jmft70026-bib-0050] Khoury, B. , B. Knäuper , F. Pagnini , N. Trent , A. Chiesa , and K. Carrière . 2017. “Embodied Mindfulness.” Mindfulness 8, no. 5: 1160–1171. 10.1007/s12671-017-0700-7.

[jmft70026-bib-0051] Khoury, B. , M. Lesage , and A. Kasprzyk , et al. 2025. “Embodied and Embedded Mindfulness and Compassion Framework.” Mindfulness 1: 02561‐4. 10.1007/s12671-025-02561-4.

[jmft70026-bib-0052] Khoury, B. , R. C. Vergara , and C. Spinelli . 2022. “Interpersonal Mindfulness Questionnaire: Scale Development and Validation.” Mindfulness 13: 1007–1031. 35308644 10.1007/s12671-022-01855-1 PMC8924575

[jmft70026-bib-0053] Kiken, L. G. , E. L. Garland , K. Bluth , O. S. Palsson , and S. A. Gaylord . 2015. “From a State to a Trait: Trajectories of State Mindfulness in Meditation During Intervention Predict Changes in Trait Mindfulness.” Personality and Individual Differences 81: 41–46. 10.1016/j.paid.2014.12.044. 25914434 PMC4404745

[jmft70026-bib-0054] Kim, J. H. 2019. “Multicollinearity and Misleading Statistical Results.” Korean Journal of Anesthesiology 72, no. 6: 558–569. 10.4097/kja.19087. 31304696 PMC6900425

[jmft70026-bib-0055] Kimmes, J. G. , M. E. Jaurequi , R. W. May , S. Srivastava , and F. D. Fincham . 2018. “Mindfulness in the Context of Romantic Relationships: Initial Development and Validation of the Relationship Mindfulness Measure.” Journal of Marital and Family Therapy 44, no. 4: 575–589. 10.1111/jmft.12296. 29073322

[jmft70026-bib-0056] Kimmes, J. G. , M. E. Jaurequi , K. Roberts , V. W. Harris , and F. D. Fincham . 2020. “An Examination of the Association Between Relationship Mindfulness and Psychological and Relational Well‐Being in Committed Couples.” Journal of Marital and Family Therapy 46, no. 1: 30–41. 10.1111/jmft.12388. 31162689

[jmft70026-bib-0057] Kimmes, J. G. , Y. Zheng , K. L. Morris , C. G. Marroquin , M. Rudaz , and D. K. Smedley . 2025. “You Are Not Fully Present With Me: How Own and Perceived Partner Mindfulness Shape Relationship Outcomes.” Journal of Family Psychology 39, no. 1: 1–10. 10.1037/fam0001290. 39666530

[jmft70026-bib-0058] Kolacz, J. , K. K. Kovacic , and S. W. Porges . 2019. “Traumatic Stress and the Autonomic Brain‐Gut Connection in Development: Polyvagal Theory as an Integrative Framework for Psychosocial and Gastrointestinal Pathology.” Developmental Psychobiology 61, no. 5: 796–809. 10.1002/dev.21852. 30953358

[jmft70026-bib-0059] Kowsar, I. , S. B. Rabbani , and M. D. Samad . 2024. “Attention‐Based Imputation of Missing Values In Electronic Health Records Tabular Data.” Proceedings. IEEE International Conference on Healthcare Informatics 2024: 177–182. 10.1109/ICHI61247.2024.00030. 39387063 PMC11463999

[jmft70026-bib-0060] Kozlowski, A. 2013. “Mindful Mating: Exploring the Connection between Mindfulness and Relationship Satisfaction.” Sexual and Relationship Therapy 28: 92–104. 10.1080/14681994.2012.748889.

[jmft70026-bib-0061] Lau, M. A. , S. R. Bishop , Z. V. Segal , et al. 2006. “The Toronto Mindfulness Scale: Development and Validation.” Journal of Clinical Psychology 62, no. 12: 1445–1467. 10.1002/jclp.20326. 17019673

[jmft70026-bib-0062] Le, B. , N. L. Dove , C. R. Agnew , M. S. Korn , and A. A. Mutso . 2010. “Predicting Nonmarital Romantic Relationship Dissolution: A Meta‐Analytic Synthesis.” Personal Relationships 17, no. 3: 377–390. 10.1111/j.1475-6811.2010.01285.x.

[jmft70026-bib-0063] Leavitt, C. E. , E. S. Lefkowitz , and E. A. Waterman . 2019. “The Role of Sexual Mindfulness in Sexual Wellbeing, Relational Wellbeing, and Self‐Esteem.” Journal of Sex & Marital Therapy 45, no. 6: 497–509. 10.1080/0092623X.2019.1572680. 30714489 PMC6640099

[jmft70026-bib-0064] Lebow, J. , and D. K. Snyder . 2022. “Couple Therapy in the 2020s: Current Status and Emerging Developments.” Family Process 61, no. 4: 1359–1385. 10.1111/famp.12824. 36175119 PMC10087549

[jmft70026-bib-0065] LeDell, E. 2018. “useR! Machine Learning Tutorial.” The R User Conference 2016. https://koalaverse.github.io/machine-learning-in-R/.

[jmft70026-bib-0066] Liu, Y. , and S. Sriutaisuk . 2021. “A Comparison of FIML‐ Versus Multiple‐Imputation‐Based Methods to Test Measurement Invariance With Incomplete Ordinal Variables.” Structural Equation Modeling: A Multidisciplinary Journal 28, no. 4: 590–608. 10.1080/10705511.2021.1876520.

[jmft70026-bib-0067] Long, J. A. 2024. “jtools: Analysis and Presentation of Social Scientific Data.” The Comprehensive R Archive Network. https://cran.r-project.org/web/packages/party/index.html.

[jmft70026-bib-0068] Luberto, C. M. , N. Shinday , R. Song , et al. 2018. “A Systematic Review and Meta‐Analysis of the Effects of Meditation on Empathy, Compassion, and Prosocial Behaviors.” Mindfulness 9, no. 3: 708–724. 10.1007/s12671-017-0841-8. 30100929 PMC6081743

[jmft70026-bib-0069] May, C. J. , B. D. Ostafin , and E. Snippe . 2020. “Mindfulness Meditation Is Associated With Decreases in Partner Negative Affect in Daily Life.” European Journal of Social Psychology 50, no. 1: 35–45. 10.1002/ejsp.2599.

[jmft70026-bib-0070] McClelland, S. I. 2011. “Who Is the ‘Self’ in Self Reports of Sexual Satisfaction? Research and Policy Implications.” Sexuality Research and Social Policy 8: 304–320. 10.1007/s13178-011-0067-9.

[jmft70026-bib-0071] McGill, J. , F. Adler‐Baeder , and L. Burke . 2022. “The Mindfulness in Couple Relationships Scale: Development and Validation.” Mindfulness 13, no. 9: 2299–2314. 10.1007/s12671-022-01957-w.

[jmft70026-bib-0072] McGill, J. , F. Adler‐Baeder , and P. Rodriguez . 2016. “Mindfully in Love: A Meta‐Analysis of the Association Between Mindfulness and Relationship Satisfaction.” Journal of Human Sciences and Extension 4, no. 1: 89–101. 10.54718/DDCA4089.

[jmft70026-bib-0073] McNulty, J. K. , C. A. Wenner , and T. D. Fisher . 2016. “Longitudinal Associations Among Relationship Satisfaction, Sexual Satisfaction, and Frequency of Sex in Early Marriage.” Archives of Sexual Behavior 45, no. 1: 85–97. 10.1007/s10508-014-0444-6. 25518817 PMC4472635

[jmft70026-bib-0074] Meyers, L. S. , G. Gamst , and A. J. Guarino . 2017. Applied Multivariate Research: Design and Interpretation. Sage.

[jmft70026-bib-0075] Morin, L. , J. C. Laurin , M. Doucerain , and S. Grégoire . 2024. “A Multilevel Diary and Dyadic Study Exploring the Link Between New Parents’ Mindfulness and Relationship Satisfaction.” Mindfulness 15, no. 9: 2330–2346. 10.1007/s12671-024-02437-z.

[jmft70026-bib-0076] Morton, L. , N. Cogan , J. Kolacz , et al. 2021. Neuroception of Psychological Safety Scale (NPSS) Manual and Scoring Guide. Traumatic Stress Research Consortium.

[jmft70026-bib-0077] Morton, L. , N. Cogan , J. Kolacz , et al. 2024. “A New Measure of Feeling Safe: Developing Psychometric Properties of the Neuroception of Psychological Safety Scale (NPSS).” Psychological Trauma: Theory, Research, Practice, and Policy 16, no. 4: 701–708. 10.1037/tra0001313. 35849369

[jmft70026-bib-0078] Newcombe, B. C. , and A. D. Weaver . 2016. “Mindfulness, Cognitive Distraction, and Sexual Well‐Being in Women.” Canadian Journal of Human Sexuality 25, no. 2: 99–108. 10.3138/cjhs.252-A3.

[jmft70026-bib-0079] Newman, M. L. and Roberts, N. A. , ed. 2013. Health and Social Relationships: The Good, The Bad, and The Complicated. American Psychological Association.

[jmft70026-bib-0080] Nguyen, M. 2020. A Guide on Data Analysis: From Basics to Causal Inference. Bookdown.

[jmft70026-bib-0081] Olendzki, A. 2014. “From Early Buddhist Traditions to Western Psychological Science.” In The Wiley Blackwell Handbook of Mindfulness, edited by A. le , C. T. Ngnoumen , and E. J. Langer , I and II, 58–73. Wiley Blackwell. 10.1002/9781118294895.ch4.

[jmft70026-bib-0082] Orth, U. 2013. “How Large Are Actor and Partner Effects of Personality on Relationship Satisfaction? The Importance of Controlling for Shared Method Variance.” Personality and Social Psychology Bulletin 39, no. 10: 1359–1372. 10.1177/0146167213492429. 23798373

[jmft70026-bib-0083] Pepping, C. A. , T. J. Cronin , A. Lyons , and J. G. Caldwell . 2018. “The Effects of Mindfulness on Sexual Outcomes: The Role of Emotion Regulation.” Archives of Sexual Behavior 47, no. 6: 1601–1612. 10.1007/s10508-017-1127-x. 29453643

[jmft70026-bib-0084] Petrocchi, N. , and S. Cheli . 2019. “The Social Brain and Heart Rate Variability: Implications for Psychotherapy.” Psychology and Psychotherapy: Theory, Research and Practice 92, no. 2: 208–223. 10.1111/papt.12224. 30891894

[jmft70026-bib-0085] Pommier, E. , K. D. Neff , and I. Tóth‐Király . 2020. “The Development and Validation of the Compassion Scale.” Assessment 27: 21–39. 10.1177/1073191119874108. 31516024

[jmft70026-bib-0086] Porges, S. W. 1995. “Orienting in a Defensive World: Mammalian Modifications of Our Evolutionary Heritage: A Polyvagal Theory.” Psychophysiology 32: 301–318. 10.1111/j.1469-8986.1995.tb01213.x. 7652107

[jmft70026-bib-0087] Porges, S. W. 2004. “Neuroception: A Subconscious System for Detecting Threats and Safety.” Zero to Three 24, no. 5: 19–24.

[jmft70026-bib-0088] Porges, S. W. 2011. The Polyvagal Theory: Neurophysiological Foundations of Emotions, Attachment, Communication, Self‐regulation. W. W. Norton & Co.

[jmft70026-bib-0089] Porges, S. W. 2017. The Pocket Guide to The Polyvagal Theory: The Transformative Power of Feeling Safe. W W Norton & Co.

[jmft70026-bib-0090] Porges, S. W. , and C. S. Carter . 2017. “Polyvagal Theory and the Social Engagement System: Neurophysiological Bridge Between Connectedness And Health.” In Complementary and Integrative Treatments in Psychiatric Practice, edited by P. L. Gerbarg , P. R. Muskin , and R. P. Brown . American Psychiatric Association.

[jmft70026-bib-0091] Porges, S. W. and Dana, D. , ed. 2018. Clinical Applications of The Polyvagal Theory: The Emergence of Polyvagal‐informed Therapies. W. W. Norton & Co.

[jmft70026-bib-0092] Pratscher, S. D. , P. K. Wood , L. A. King , and B. A. Bettencourt . 2019. “Interpersonal Mindfulness: Scale Development and Initial Construct Validation.” Mindfulness 10, no. 6: 1044–1061. 10.1007/s12671-018-1057-2.

[jmft70026-bib-0093] Proulx, C. M. , H. M. Helms , and C. Buehler . 2007. “Marital Quality and Personal Well‐Being: A Meta‐Analysis.” Journal of Marriage and Family 69, no. 3: 576–593.

[jmft70026-bib-0094] Quinn‐Nilas, C. 2020a. “Relationship and Sexual Satisfaction: A Developmental Perspective on Bidirectionality.” Journal of Social and Personal Relationships 37, no. 2: 624–646. 10.1177/0265407519876018.

[jmft70026-bib-0095] Quinn‐Nilas, C. 2020b. “Self‐Reported Trait Mindfulness and Couples' Relationship Satisfaction: A Meta‐Analysis.” Mindfulness 11: 835–848. 10.1007/s12671-020-01303-y.

[jmft70026-bib-0096] R Core Team . 2023. “R: A Language and Environment for Statistical Computing [Software].” Vienna: R Foundation for Statistical Computing. https://www.R-project.org/.

[jmft70026-bib-0097] Ryland, S. , L. N. Johnson , and J. C. Bernards . 2022. “Honoring Protective Responses: Reframing Resistance in Therapy Using Polyvagal Theory.” Contemporary Family Therapy 44: 267–275. 10.1007/s10591-021-09584-8.

[jmft70026-bib-0098] Sánchez‐Fuentes, M. M. , P. Santos‐Iglesias , and J. C. Sierra . 2014. “A Systematic Review of Sexual Satisfaction.” International Journal of Clinical and Health Psychology 14, no. 1: 67–75. 10.1016/S1697-2600(14)70038-9.

[jmft70026-bib-0099] Siegel, J. P. 2014. “The Mindful Couple.” Clinical Social Work Journal 42: 282–287. 10.1007/s10615-014-0489-y.

[jmft70026-bib-0100] Smedley, D. K. , C. E. Leavitt , D. B. Allsop , M. Nance , S. L. James , and E. K. Holmes . 2021. “Mindfulness and Sexual Mindfulness as Moderators between Conflict Resolution and Sexual and Relationship Satisfaction.” Journal of Sex & Marital Therapy 47, no. 8: 814–828. 10.1080/0092623X.2021.1958962. 34472422

[jmft70026-bib-0101] Spreng*, R. N. , M. C. McKinnon* , R. A. Mar , and B. Levine . 2009. “The Toronto Empathy Questionnaire: Scale Development and Initial Validation of a Factor‐Analytic Solution to Multiple Empathy Measures.” Journal of Personality Assessment 91, no. 1: 62–71. 10.1080/00223890802484381. 19085285 PMC2775495

[jmft70026-bib-0102] Stanton, S. C. E. , A. P. S. Chan , and T. Gazder . 2021. “Mindfulness, Perceived Partner Responsiveness, and Relationship Quality: A Dyadic Longitudinal Mediation Model.” Journal of Social and Personal Relationships 38, no. 11: 3310–3332. 10.1177/02654075211030327.

[jmft70026-bib-0103] Statistics Canada . 2024. “Canada at a Glance, 2023: 2SLGBTQ+ Population.” https://www150.statcan.gc.ca/n1/pub/12-581-x/2023001/Section6-eng.htm.

[jmft70026-bib-0104] Strobl, C. , J. Malley , and G. Tutz . 2009. “An Introduction to Recursive Partitioning: Rationale, Application, and Characteristics of Classification and Regression Trees, Bagging, and Random Forests.” Psychological Methods 14, no. 4: 323–348. 19968396 10.1037/a0016973 PMC2927982

[jmft70026-bib-0105] Vabalas, A. , E. Gowen , E. Poliakoff , and A. J. Casson . 2019. “Machine Learning Algorithm Validation with a Limited Sample Size.” PLoS One 14, no. 11: e0224365. 10.1371/journal.pone.0224365. 31697686 PMC6837442

[jmft70026-bib-0106] Vatcheva, K. P. , M. Lee , J. B. McCormick , and M. H. Rahbar . 2016. “Multicollinearity in Regression Analyses Conducted in Epidemiologic Studies.” Epidemiology 6, no. 2: 1000227. 10.4172/2161-1165.1000227. PMC4888898 27274911

[jmft70026-bib-0107] Vowels, L. M. , and K. P. Mark . 2020. “Relationship and Sexual Satisfaction: A Longitudinal Actor–Partner Interdependence Model Approach.” Sexual and Relationship Therapy 35, no. 1: 46–59. 10.1080/14681994.2018.1441991.

[jmft70026-bib-0108] Vowels, L. M. , M. J. Vowels , and K. P. Mark . 2022. “Identifying the Strongest Self‐Report Predictors of Sexual Satisfaction Using Machine Learning.” Journal of Social and Personal Relationships 39, no. 5: 1191–1212. 10.1177/02654075211047004.

[jmft70026-bib-0109] Weiss, R. L. 1980. “Strategic Behavioral Relationship Therapy: Toward A Model For Assessment And Intervention.” In Advances In Family Intervention, Assessment And Theory, edited by J. P. Vincent , 229–271. JAI Press.

[jmft70026-bib-0110] Williamson, H. C. , J. X. Bornstein , V. Cantu , O. Ciftci , K. A. Farnish , and M. T. Schouweiler . 2022. “How Diverse Are the Samples Used to Study Intimate Relationships? A Systematic Review.” Journal of Social and Personal Relationships 39, no. 4: 1087–1109. 10.1177/02654075211053849. 35655791 PMC9159543

[jmft70026-bib-0111] Winter, F. , A. Steffan , M. Warth , B. Ditzen , and C. Aguilar‐Raab . 2021. “Mindfulness‐Based Couple Interventions: A Systematic Literature Review.” Family Process 60, no. 3: 694–711. 10.1111/famp.12683. 34114656

[jmft70026-bib-0112] Wood, N. D. , D. R. Crane , G. B. Schaalje , and D. D. Law . 2005. “What Works for Whom: A Meta‐Analytic Review of Marital and Couples Therapy in Reference to Marital Distress.” American Journal of Family Therapy 33, no. 4: 273–287.

[jmft70026-bib-0113] Porges, S. W. 1998. “Love: An Emergent Property of the Mammalian Autonomic Nervous System.” Psychoneuroendocrinology 23, no. 8: 837–861. 9924740 10.1016/s0306-4530(98)00057-2

[jmft70026-bib-0114] Yeh, H.‐C. , F. O. Lorenz , K. A. S. Wickrama , R. D. Conger , and G. H. Elder . 2006. “Relationships Among Sexual Satisfaction, Marital Quality, and Marital Instability At Midlife.” Journal of Family Psychology 20, no. 2: 339–343. 16756411 10.1037/0893-3200.20.2.339

[jmft70026-bib-0115] Zacchilli, T. L. , C. Hendrick , and S. S. Hendrick . 2009. “The Romantic Partner Conflict Scale: A New Scale to Measure Relationship Conflict.” Journal of Social and Personal Relationships 26, no. 8: 1073–1096. 10.1177/0265407509347936.

