# Supplementary material for: A Machine Learning Approach for Investigating Variable Importance in Relationship and Sexual Satisfaction: The Role of Interpersonal Mindfulness and Psychological Safety
Source: J Marital Fam Ther. 2025 Apr 24;51(2):e70026. doi: 10.1111/jmft.70026 (PMC12022470; doi:10.1111/jmft.70026)
Supplement: Supplementary file 1 — Descriptive Results of Instruments Administered. [file JMFT-51-0-s001.docx]

**Supporting Information**

*Descriptive Results of Instruments Administered*

| Instrument | Mean (SD) | Median [Min, Max] | Missing |
| --- | --- | --- | --- |
| **Mindfulness** | | | |
| FFMQ Total | 51.3 (8.71) | 51.0 [19.0, 75.00] | 4 (1.1%) |
| FFMQ Observe | 10.8 (2.41) | 11.0 [3.00, 15.00] |  |
| FFMQ Describe | 10.8 (2.66) | 11.0 [3.00, 15.00] |  |
| FFMQ Acting with Awareness | 8.96 (2.59) | 9.00 [3.00, 15.00] | 1 (0.3%) |
| FFMQ Non-Judging | 10.9 (3.07) | 11.0 [3.00, 15.00] |  |
| FFMQ Non-Reactivity | 9.84 (2.53) | 10.0 [3.00, 15.00] | 3 (0.8%) |
| IMS Total | 104 (13.9) | 105 [63.0, 135] | 7 (2.0%) |
| IMS Presence | 25.0 (5.07) | 25.0 [9.00, 35.0] | 1 (0.3%) |
| IMS Awareness of Self and Others | 40.8 (6.16) | 41.0 [20.0, 50.00] | 2 (0.6%) |
| IMS Nonjudgmental Acceptance | 15.9 (2.39) | 16.0 [6.00, 20.00] | 3 (0.8%) |
| IMS Nonreactivity | 22.8 (3.74) | 23.0 [13.0, 30.00] | 3 (0.8%) |
| IMQ Being Caught in the Mind | 2.69 (0.79) | 2.60 [1.00, 5.00] | 1 (0.3%) |
| IMQ Attention to and Awareness of Others | 4.23 (0.76) | 4.33 [1.00, 5.00] | 4 (1.1%) |
| IMQ Body-Anchored Presence | 3.62 (0.92) | 3.67 [1.00, 5.00] | 2 (0.6%) |
| IMQ Mindful Responding | 3.96 (0.56) | 4.00 [1.60, 5.00] | 2 (0.6%) |
| MCRS Total | 5.78 (0.67) | 5.84 [2.55, 7.00] | 3 (0.8%) |
| MCRS Non-judging | 5.57 (0.98) | 5.75 [2.25, 7.00] |  |
| MCRS Patience | 5.70 (0.90) | 5.75 [1.00, 7.00] |  |
| MCRS Beginner’s Mind | 5.94 (0.95) | 6.00 [1.00, 7.00] | 1 (0.3%) |
| MCRS Trust of Self | 6.04 (0.90) | 6.00 [1.00, 7.00] | 1 (0.3%) |
| MCRS Non-Striving | 6.02 (0.90) | 6.33 [1.00, 7.00] |  |
| MCRS Acceptance | 5.97 (0.82) | 6.00 [2.33, 7.00] |  |
| MCRS Letting Go | 4.95 (1.29) | 5.00 [1.00, 7.00] |  |
| MCRS Noticing | 6.08 (0.62) | 6.14 [2.71, 7.00] | 1 (0.3%) |
| **Social and Couple-Related** | | | |
| CSI Total | 126 (26.0) | 131 [21.0, 161] | 62 (17.4%) |
| NSSS Total | 44.6 (10.10) | 46.0 [12.0, 60.00] | 17 (4.8%) |
| NSSS Ego-Centered | 22.5 (5.37) | 23.0 [6.00, 30.00] | 10 (2.8%) |
| NSSS Partner/Activity-Centered | 22.0 (5.56) | 23.0 [6.00, 30.00] | 12 (3.4%) |
| NPSS Total | 4.18 (0.62) | 4.21 [1.14, 5.00] | 7 (2.0%) |
| NPSS Social Engagement | 4.24 (0.71) | 4.36 [1.00, 5.00] | 4 (1.1%) |
| NPSS Compassion | 4.38 (0.64) | 4.57 [1.00, 5.00] |  |
| NPSS Bodily Sensations | 3.92 (0.82) | 4.00 [1.00, 5.00] | 3 (0.8%) |
| RPCS Compromise | 58.1 (10.90) | 60.0 [14.0, 70.00] | 6 (1.7%) |
| RPCS Avoidance | 9.81 (3.40) | 10.0 [3.00, 15.00] | 4 (1.1%) |
| RPCS Interactional Reactivity | 10.5 (4.97) | 9.00 [6.00, 29.00] | 1 (0.3%) |
| RPCS Separation | 15.2 (5.20) | 16.0 [5.00, 25.0] | 5 (1.4%) |
| RPCS Domination | 12.7 (5.87) | 12.0 [6.00, 30.0] | 2 (0.6%) |
| RPCS Submission | 12.4 (5.55) | 12.0 [5.00, 25.0] | 5 (1.4%) |
| CS Total | 4.21 (0.52) | 4.25 [1.69, 5.00] | 8 (2.2%) |
| CS Kindness | 4.31 (0.68) | 4.50 [1.00, 5.00] | 3 (0.8%) |
| CS Common Humanity | 4.18 (0.66) | 4.25 [1.75, 5.00] | 4 (1.1%) |
| CS Mindfulness | 4.28 (0.61) | 4.25 [1.50, 5.00] | 1 (0.3%) |
| CS Indifference | 1.96 (0.84) | 1.75 [1.00, 5.00] | 1 (0.3%) |
| TEQ Total | 50.2 (8.35) | 51.0 [19.0, 64.00] | 7 (2.0%) |
| **Emotional Wellbeing** | | | |
| DASS Depression | 10.0 (10.2) | 8.00 [0, 42.00] | 1 (0.3%) |
| DASS Anxiety | 8.16 (9.08) | 4.00 [0, 42.00] | 3 (0.8%) |
| DASS Stress | 13.1 (9.81) | 12.0 [0, 42.00] | 8 (2.2%) |
| DERS Total | 2.11 (0.66) | 2.06 [1.00, 4.39] | 3 (0.8%) |
| DERS Strategies | 1.98 (0.95) | 1.67 [1.00, 5.00] | 3 (0.8%) |
| DERS Non-Acceptance | 2.22 (1.07) | 2.00 [1.00, 5.00] | 1 (0.3%) |
| DERS Impulse | 1.60 (0.84) | 1.33 [1.00, 5.00] |  |
| DERS Goals | 2.93 (1.11) | 2.67 [1.00, 5.00] |  |
| DERS Awareness | 2.02 (0.80) | 2.00 [1.00, 4.67] |  |
| DERS Clarity | 1.92 (0.89) | 1.67 [1.00, 5.00] |  |

*Note.* The table presents the descriptives and the frequency of missing values among variables of interest. Overall (N = 356).
